# High HPgV replication is associated with improved surrogate markers of HIV progression

**DOI:** 10.1371/journal.pone.0184494

**Published:** 2017-09-14

**Authors:** Gibran Horemheb-Rubio, Pilar Ramos-Cervantes, Hugo Arroyo-Figueroa, Santiago Ávila-Ríos, Claudia García-Morales, Gustavo Reyes-Terán, Galileo Escobedo, Gloria Estrada, Trinidad García-Iglesias, Nayeli Muñoz-Saucedo, David Kershenobich, Patricia Ostrosky-Wegman, Guillermo M. Ruiz-Palacios

**Affiliations:** 1 Department of Infectious Diseases, Instituto Nacional de Ciencias Médicas y Nutrición Salvador Zubirán, Mexico City, Mexico; 2 Infectious Diseases Research Center, Instituto Nacional de Enfermedades Respiratorias, Mexico City, Mexico; 3 Liver Pancreas and Intestinal Motility Laboratory, Hospital General de México, Facultad de Medicina, Universidad Nacional Autónoma de México, Mexico City, Mexico; 4 Blood Bank, Hospital General de México, Mexico City, Mexico; 5 Immnuology Laboratory, Centro Universitario de Ciencias de la Salud, Universidad de Guadalajara, Guadalajara, Jalisco, Mexico; 6 Instituto Nacional de Ciencias Médicas y Nutrición Salvador Zubirán, Mexico City, Mexico; 7 Biomedical Research Institute, Universidad Nacional Autónoma de México, Mexico City, Mexico; University of Cincinnati College of Medicine, UNITED STATES

## Abstract

**Background:**

Human Pegivirus (HPgV) may have a beneficial effect on HIV disease progression in co-infected patients; however, the virologic characteristics of this infection are not well defined. In this study, we determined HPgV viremia prevalence in Mexico and provide new insights to understand HPgV infection and HPgV/HIV co-infection.

**Methods:**

We analyzed and quantified 7,890 serum samples for HPgV viremia by One-Step RT-Real-Time PCR, 6,484 from healthy blood donors and 1,406 from HIV-infected patients. Data on HIV progression were obtained from patients’ records. HPgV genotyping was performed in 445 samples by nested PCR of the 5’URT region. Finite Mixture Models were used to identify clustering patterns of HPgV viremia in blood donors and co-infected antiretroviral (ART)-naïve patients.

**Results:**

HPgV was detected in 2.98% of blood donors and 33% of HIV patients, with a wide range of viral loads. The most prevalent genotypes were 3 (58.6%)and 2 (33.7%). HPgV viral loads from healthy blood donors and HPgV/HIV+ ART-naïve co-infected patients were clustered into two component distributions, low and high, with a cut-off point of 5.07log_10_ and 5.06log_10_, respectively. High HPgV viremia was associated with improved surrogate markers of HIV infection, independent of the estimated duration of HIV infection or HIV treatment.

**Conclusions:**

HPgV prevalence in Mexico was similar to that reported for other countries. The prevalent genotypes could be related to Mexico’s geographic location and ethnicity, since genotype 2 is frequent in the United States and Europe and genotype 3 in Asia and Amerindian populations. HPgV viral load demonstrated two patterns of replication, low and high. The more pronounced beneficial response observed in co-infected patients with high HPgV viremia may explain discrepancies found between other studies. Mechanisms explaining high and low HPgV replication should be explored to determine whether the persistently elevated replication depends on host or viral factors.

## Introduction

Human Pegivirus (HPgV), formerly GB virus C (GBV-C), was considered non-pathogenic, although this concept has changed due to its association with non-Hodgkin’s lymphoma [1,2]. HPgV is classified in the genus Pegivirus within the *Flaviviridae* and it is the most prevalent flavivirus in the world [3]. The prevalence of HPgV viremia is 1–5% among healthy blood donors, although higher prevalences have been found in developing countries (up to 18%) [4] Co-infection with HPgV occurs in 20–40% of HIV patients; both viruses share transmission routes: blood transfusion, sexual and vertical [5–8]. HPgV has 7 genotypes, geographically distributed: genotypes 1 and 5 predominate in Africa, genotype 2 in Europe and North America, genotype 3 in Asia and Amerindian populations, genotype 4 in the Philippines and Mainland Southeast Asia, genotype 6 in China and Japan, and 7 in China, although there is controversy in whether genotypes 6 and 7 are variations of genotype 4 [9–16]. Several studies have shown that co-infected patients have slower HIV progression, increased survival time, and decreased vertical HIV transmission, all of these described as HPgV beneficial effects. However, there are discrepancies among studies on the frequency of these effects, which have been extensively reviewed [17–20].

This research aimed to understand HPgV infection and HPgV/HIV co-infection in Mexico by studying the conditions under which these beneficial effects occur *in vivo*. We assessed HPgV viremia prevalence and viral load by One-Step RT-Real-Time PCR in healthy blood donors and HIV patients, analysed HPgV viral load distribution within the positive population, and determined the effect of HPgV on HIV viral load, CD4^+^ cell count, and CD4^+^/CD8^+^ ratio in HIV-positive antiretroviral (ART)-naïve patients.

## Materials and methods

### Serum samples' collection

We tested 7,890 serum samples for HPgV viremia, 6,484 from healthy blood donors and 1,406 from HIV-positive patients (770 ART-naïve and 636 on HAART). We obtained samples from blood donors during the periods of August–December 2009, March–June 2010, and January–August 2015, at the blood banks of four referral hospitals from central and western Mexico: National Institute of Medical Sciences and Nutrition Salvador Zubiran (INCMNSZ) and General Hospital of Mexico in Mexico City; Old Civil Hospital Fray Antonio Alcalde and New Civil Hospital Juan I. Menchaca in Guadalajara, Jalisco. Written informed consent was signed by blood donors before sample collection. The study was approved by the research and ethics committees of each institution: Comité de Ética en Investigación del Instituto Nacional de Ciencias Médicas y Nutrición Salvador Zubirán, Comité de Ética en Investigación del Hospital General de México, Comité de Ética en Investigación del Hospital Fray Antonio Alcalde, and Comité de Ética del Hospital Civil Juan I. Menchaca.

We collected 5 ml of blood from healthy blood donors at donation time. Blood samples were centrifuged at 3000xg and serum aliquots were made and stored at -70°C until tested. We obtained serum samples from HAART patients from the INCMNSZ HIV Clinic repository, collected from February 2004 to May 2010. Samples from HIV-positive ART-naïve patients, collected from October 2007 to July 2013, were obtained from the Infectious Disease Research Centre at the National Institute of Respiratory Diseases (INER-CIENI) repository in Mexico City.

### Serum samples pooling and nucleic acid extraction

**Fig 1:**
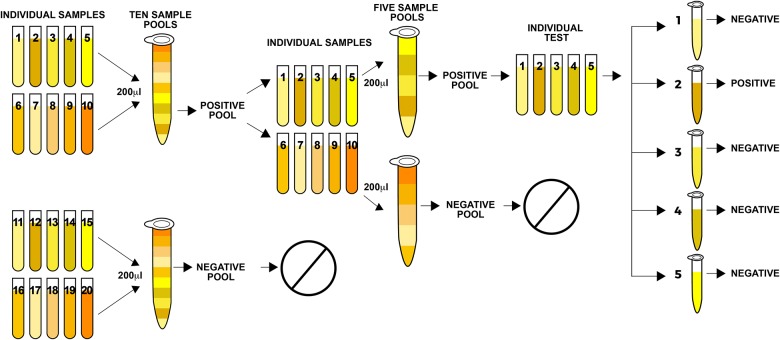
Diagram of pooling method for serum samples from blood donors. The diagram shows the construction of sample pools and analysis for HPgV viremia in healthy blood donors. Positive samples from the pool were re-tested in smaller pools each time until we detected the positive individual donor sample.

To test healthy blood donor samples for HPgV infection, we formed pools to save reagents [21,22], as follows (Fig 1): 200ul from each sample were mixed in one tube to make pools of 10 samples each; 500ul were taken from each pool and processed by an automatic nucleic acid extraction system (NucliSENS^®^easyMag^®^, Biomerieux, France) according to the manufacturer’s instructions. Nucleic acids were tested for HPgV viremia by One-Step RT-Real-Time PCR as described in the following section. We performed a second round of pools, mixing 200ul from the samples that were positive to HPgV in the first pool test to make up new 5-samples pools, which were re-tested. Then, to identify HPgV viremic donors we tested individually the positive samples from the second round of pools. Samples from HIV patients were tested one at a time, and viral load was quantified in the positive samples.

### HPgV detection, quantification and genotyping

#### HPgV detection

We tested nucleic acids by One-Step RT-Real-Time PCR with a lower detection of 50 copies per ml of serum, as follows: retro-transcription at 50°C for 30 min, polymerase activation at 95°C for 2 min, 40 cycles of primer annealing at 95°C for 15 sec, and elongation-detection at 55°C for 1 min. Selected primers correspond to the 5’ untranslated region (103–163nt) according to GenBank accession number AF121950: sense primer (103-122nt) 5’- GGC CAA AAG GTG GTG GAT GG -3’ [10]; antisense primer (188-163nt) 5’- CTT AAG ACC CAC CTA TAG TGG CTA CC -3’; and the probe (131-158nt) 5’- (FAM) TGA CCG GGA TTT ACG ACC TAC CAA CCC T (TAMRA) -3’ [23].

#### HPgV quantification

The One-Step RT-Real-Time PCR described above was used for HPgV quantification of the individual positive samples. A puc57 plasmid containing one copy of the PCR amplified 5’UTR HPgV region fragment (85-362nt) according to GenBank accession number AF121950, was used as a standard curve reference in concentrations ranging from 10^9^ to 10^2^ copies.

#### HPgV genotyping

Genotyping was performed in 445 HPgV-positive samples. We extracted nucleic acids by standard procedures and performed nested PCR. Briefly, the first pair of primers amplified the HPgV 5’ untranslated (5’UTR) region fragment (139-400nt) according to GenBank accession number U44402; a second PCR was done to differentiate genotypes 1–4 by molecular weight as described by Naito et al. [9]. Capillary sequencing of the 5’UTR region fragment was performed to test the samples that did not amplify or those with inconclusive results due to the presence of more than one genotype. HPgV genotype was established according to alignment with the reference sequences corresponding to genotypes 1 to 7 [24], GenBank accession numbers: U59547 and AF131116, genotype 1; AB003289, U45966, U44402, U59520, U59521, U59519, AF131118, AY196904, AF104403, and U59518, genotype 2a; U59529, U59532, U59530, U59531, and U59533, genotype 2b; D87711, AB003288, D87251, D87710, AB008335, D87714, D87708, D87713, D87712, AB003290, D87709, and D90601, genotype 3; AB021287, HQ331172, and AB018667, genotype 4; AF131111, and AF131112, genotype 5; AB003292, genotype 6; HQ331234, HQ331233, and HQ331235, genotype 7. Phylogenetic and molecular evolutionary analyses were done using MEGA version 6 [25].

### HIV quantification, CD4^+^ cell counts, CD4^+^/CD8^+^ ratio, and assessment of HIV infection time

HIV viral loads, CD4^+^ cell counts, CD4^+^/CD8^+^ ratio, and HIV infection time (defined as recent [<6 months] and late [≥6 months]) of the ART-naïve patients were previously determined and shared by CIENI-INER. Criteria to consider recent infections were: individuals with <1 year of diagnosis, CD4+ T cell counts ≥200 cells/μL, plasma HIV viral load >400 RNA copies/ml, and HIV-1 Lag-Avidity EIA (Sedia, USA) ODn ≤1.5 (95% CI 118–142) with a seroconversion period window of 130 days. All samples were tested in duplicate. Individuals who did not meet these criteria were considered as having a late infection [26,27].

### Statistical analysis

#### HPgV viral load heterogeneity

Due to the wide HPgV viral load heterogeneity among blood donors and the HIV-positive ART-naïve group, we verified the existence of clusters dependant of HPgV viral load. For this purpose, we performed a Finite Mixture Model (FMM) in R software (Version 3.1.3 released 2015-03-09), fitting a Gaussian distribution in both HPgV-positive populations (blood donors and HIV-positive ART-naïve) separately by using NormalmixEM and plot.mixEM functions contained in mixtools package (Version 1.0.3 released 2015-04-18) [28]. The optimal cut-off point is the value where the probability density curves of both clusters cross.

The NormalmixEM uses the Expectation-Maximization (EM) algorithm to determine, by repeating iterations, the joining likelihood between each sample’s viral loads until the model converges. To improve the model and ensure that the analyses of HPgV viral load distribution of both populations (blood donors and HIV-positive ART-naïve) where comparable, we decided to specify the initial values of lambda and sigma instead of leaving them unsupervised [29,30].

The initial sigma value in the model was 1.4 for both populations, calculated as the standard deviation (SD) of HPgV viral load distributions within the entire healthy blood donors HPgV-positive population (SD = 1.4). By selecting only this population to determine the initial sigma value (standard deviation, determined as the initial factor for the FMM model for both groups), we avoided the influence of HIV infection on the SD of the whole population analysed. The initial lambda value (proportion of the subjects in each formed subgroup) used was 0.5, defined as an initial proportion for the formed clusters. This approach helped us reduce the number of iterations until the model converged, and increase the loglik at the estimate number [31]. Co-infected HAART patients were excluded to avoid treatment as a confounding factor. We then compared by Student’s t-Test the means of HPgV viral load of HPgV -low and HPgV -high, controlling with HPgV genotypes 2 and 3.

#### Effect of HPgV viral load on surrogate markers of HIV progression

We compared the means of the following parameters: HIV viral load, CD4+ cell counts [32,33], and CD4^+^/CD8^+^ ratio [34]. These comparisons were performed according to HPgV major prevalent genotypes (2 and 3) and HPgV condition (HPgV-negative and HPgV-positive) by Student’s *t*-Test, one-way ANOVA and Bonferroni multiple-comparison test in overall ART-naïve patient population and in the subsequent division into recent (< 6 months) and late (≥ 6 months) HIV infection time groups. Analyses and graphs were made in STATA and SIGMAPLOT software.

## Results

### HPgV viremia, viral load and genotyping

One of the aims of this study was to characterise HPgV infection in Mexico. We determined the prevalence of HPgV viremia, viral load and genotype in healthy blood donors and HIV-positive patients by One-Step RT-Real Time PCR (S1 Fig). HPgV viremia was detected in 191 (2.94%) of 6,484 samples from blood donors and 473 (33.64%) of 1,406 samples from all HIV-positive patients included (ART-experienced and ART-naïve). We found a wide range of viral loads in positive samples, 10^2^−10^8^ genome equivalents (GE). HPgV genotype was determined in 445 samples (72/191 from healthy blood donors and 373/473 HIV-positive patients from which 165/180 were ART-experienced and 208/293 were ART-naïve). The most prevalent genotypes were 3 (261, 58.6%) and 2 (150, 33.7%). We found coinfection with genotypes 2/3 (30, 6.7%), from which 20 (4.5%) were genotype 2a/3 and 10 (2.2%) were 2b/3 (S5 Fig). We also found a low prevalence of genotypes 1 and 4 (with 2 samples each, 0.45%). There were no significant differences in means of HIV viral load (p>0.05), CD4+ cell counts (p>0.05), CD4+/CD8+ ratio (p>0.05) and HPgV viral load (p>0.05) from HIV positive ART-naïve patients, between major HPgV genotypes 2 and 3 in HPgV -low or HPgV -high tested by one-way ANOVA and confirmed by Bonferroni test (Table 1).

**Table 1:** Surrogate markers of HIV progression compared between genotypes 2 and 3, according to the low or high HPgV viral loads in HIV-positive ART-naïve patients.

*Condition*	HPgV—low			HPgV—high		
***Genotype***	2 (n = 23*)	3 (n = 76)	p—value	2 (n = 22)	3 (n = 54)	P—value
***Mean of HIV viral load, GE(log10)/ml***	4.98 95%CI (4.66–5.28)	4.80 95%CI (4.63–4.97)	0.33	4.70 95%CI (4.39–4.99)	4.44 95%CI (4.25–4.63)	0.15
***Mean of CD4+ cell counts***	323 95%CI (215–431)	293 95%CI (233–352)	0.63	344 95%CI (251–437)	394 95%CI (335–453)	0.37
***Mean of CD4+/CD8+ ratio***	.325 95%CI (.231 - .418)	.286 95%CI (.234 - .337)	0.46	.379 95%CI (.271 - .485)	.394 95%CI (.324 - .462)	0.82
***Mean of HPgV viral load, GE(log10)/ml***	3.35 95%CI (3.04–3.64)	3.58 95%CI (3.41–3.74)	0.18	6.42 95%CI (6.11–6.72)	6.43 95%CI (6.23–6.62)	0.96

The table shows the differences between HPgV genotypes 2 and 3 (by Student’s t-Test and Bonferroni multiple comparison test) in means of HIV viral load, CD4+ cell counts, CD4+/CD8+ ratio and HPgV viral load. HIV and HPgV viral loads are expressed as genomic equivalents (GE)/ml (log10).

*One patient (ID 667) was excluded from this part of the analysis due to his outlier CD4+ cell count. Statistical significance was considered at p<0.05.

### HIV quantification, CD4^+^ cell counts, CD4^+^/CD8^+^ ratio, and the assessment of HIV infection time

To compare the HPgV effect on surrogate markers of HIV infection, we used the previously determined results shared by CIENI-INER [26,27] of HIV viral loads, CD4^+^ cell counts and CD4^+^/CD8^+^ ratio from 770 HIV-positive ART-naïve patients and HIV infection time from 303 HIV-positive ART-naïve patients.

### HPgV viral load heterogeneity

**Fig 2:**
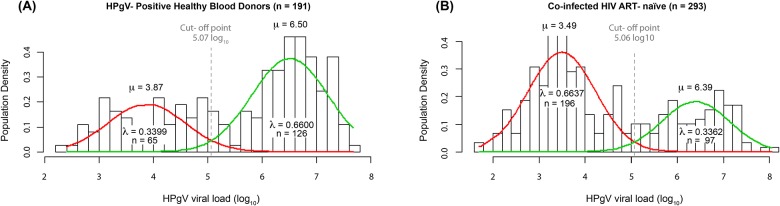
Two-component cluster mixture model fit for weighted HPgV viral load from healthy blood donors and co-infected ART-naïve patients. Density distribution (bars) of HPgV viral load (log_10_ genome equivalents) from healthy blood donors (A) and co-infected ART-naïve patients (B). Gaussian curves indicate the fitted low (red line), high (green line), mean component curves (μ), the number of patients (n) and proportion (λ) in each population component. The clusters’ cut-off point for healthy blood donors (A) and co-infected ART-naïve patients (B), is the intersection of the curves.

Our prevalence results show a wide heterogeneity of HPgV viral load. To verify the existence of subgroups, we analysed the HPgV viral load distribution patterns in healthy blood donors and co-infected ART-naïve patients (S2 Fig). By using the FMM analysis, we found that subjects were clustered into two component distributions with similar cut-off points, 5.07log_10_ for blood donors and 5.06log_10_ for ART-naïve patients. We named these clusters HPgV-low and -high conditions. Then, we determined the population density distribution of each cluster. We found that 34% of the blood donors belonged to the HPgV-low and 66% to the HPgV-high cluster (Fig 2A); while among the co-infected ART-naïve population 66.3% belonged to the HPgV-low and 33.6% to the HPgV-high clusters (Fig 2B). To assess whether HPgV viral load clustering was determined by the genotype, we analysed the genotyped samples from HIV-positive ART-naïve patients corresponding to the major prevalent HPgV genotypes (2 and 3). From these, 100 corresponded to the HPgV low-viral load cluster, from which 76 (76%) were genotype 3 and 24 (24%) were genotype 2. Of 76 genotyped samples corresponding to HPgV high- viral load cluster, 54 (71%) were genotype 3 and 22 (29%) were genotype 2.

### Effect of HPgV viral load on surrogate markers of HIV progression

**Fig 3:**
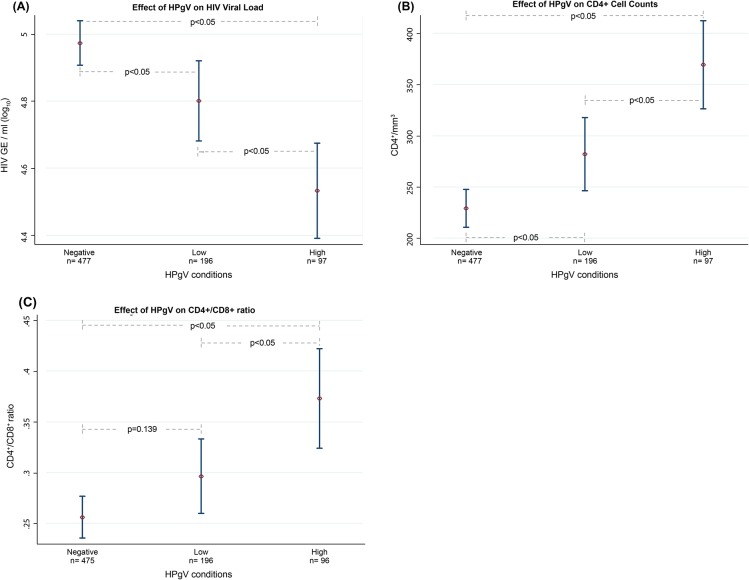
Effects of HPgV on surrogate markers of HIV progression. Each graph shows the differences between the means of: (A) HIV viral load/ml (log_10_), (B) CD4^+^ cell counts (CD4^+^/mm^3^), and (C) CD4^+^/CD8^+^ ratio, in the ART-naïve population according to their HPgV condition (-negative, -low, or -high). Significant differences are considered at p<0.05.

Studies have shown the effect of HPgV on surrogate markers of HIV progression (HIV viral load, CD4^+^ cell counts, and CD4^+^/CD8^+^ ratio); however, the majority of these studies have not considered HPgV viral load and discrepancies remain [17]. To determine the role that HPgV viral load and the cluster distribution pattern play in the effect of HPgV on HIV, we compared in the ART-naïve population the means of markers of HIV progression by one-way ANOVA, divided according to HPgV viral load conditions (-negative, -low and -high) (S3 Fig). We found statistically significant differences between means of HIV viral load (p<0.05) (Fig 3A), CD4^+^ cell counts (p<0.05) (Fig 3B) and CD4^+^/CD8^+^ ratio (p<0.05) (Fig 3C). We then used Bonferroni comparison test to verify the specific effect of each HPgV condition on HIV progression markers, using the HPgV-negative as a comparison reference. We found significantly lower means of HIV viral loads (p <0.05) in the HPgV-low (-0.17 HIV copies/ml log_10_) and HPgV-high (-0.44 HIV copies/ml log_10_) conditions; significantly higher means of CD4^+^ cell counts (p <0.05) in the HPgV-low (+53 CD4^+^/mm^3^) and HPgV-high (+140 CD4^+^/mm^3^) conditions; and significantly higher CD4^+^/CD8^+^ ratio (p <0.05) only in the HPgV-high condition (+0.11).

**Fig 4:**
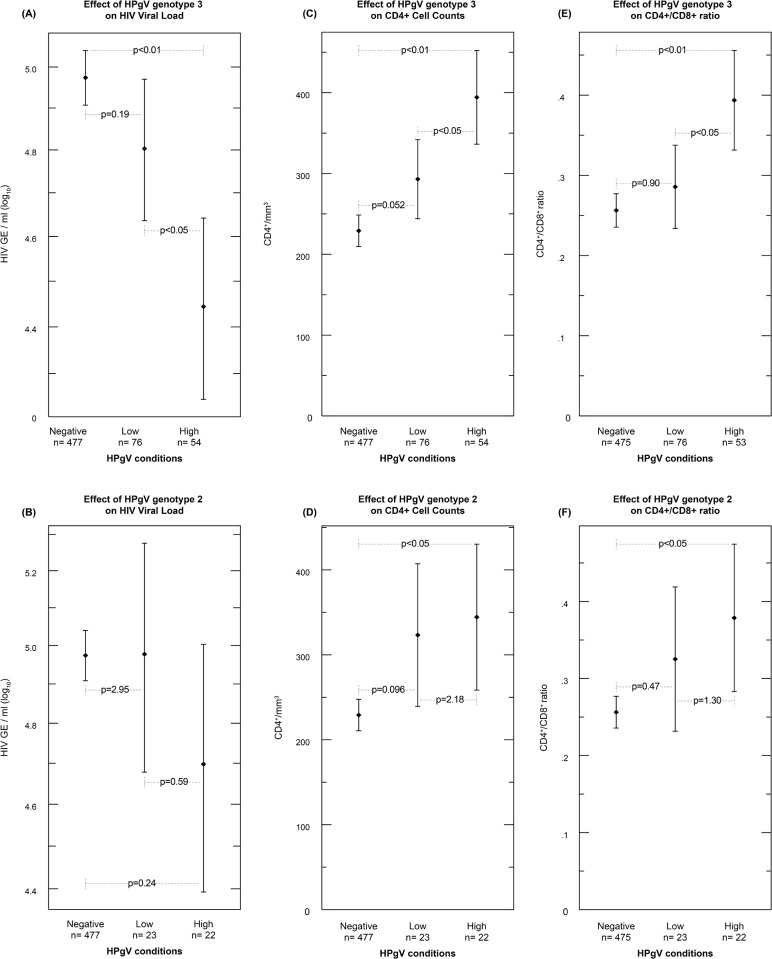
Effects of HPgV genotype (2 or 3) on surrogate markers of HIV progression. Each graph shows the differences between the means of: (A and B) HIV viral load/ml (log_10_), (C and D) CD4^+^ cell counts (CD4^+^/mm^3^), and (E and F) CD4^+^/CD8^+^ ratio, in the ART-naïve population according to their HPgV condition (-negative, -low, or -high) and genotype 3 (A, C and E) and genotype 2 (B, D and F). Significant differences are considered at p<0.05.

To determine the influence of HPgV genotype on surrogate markers of HIV progression, we compared the means of HIV viral load, CD4+ cell counts and CD4+/CD8+ ratio from HIV positive ART-naïve patients by one-way ANOVA and Bonferroni multiple comparison test between HPgV -negative -low or –high in samples corresponding to genotypes 2 or 3. We found that only genotype 3 was associated with a significant effect on all three markers of HIV progression. There was a significantly lower HIV viral load in HPgV genotype 3 –high (p<0.01, -0.53 HIV copies/ml log10) compared with HPgV –negative, and in genotype 3 –high (p<0.05, -0.36 HIV copies/ml log10) compared with genotype 3 –low (Fig 4A). When we compared means of HIV viral load in HPgV genotype 2 samples, we did not find significant differences between any of the HPgV conditions (-negative, -low and -high) (p>0.05) (Fig 4B). When comparing means of CD4+ cell counts between HPgV conditions in samples of patients with genotype 3, we found significantly higher CD4+ counts in HPgV–high (p<0.01, +166) compared with –negative; and in –high (p<0.05, +101) compared with –low (Fig 4C). We also found significantly higher CD4+ counts in samples of patients with HPgV genotype 2 –high (p<0.05, +115) compared with HPgV-negative (Fig 4D). Furthermore, significantly higher means of CD4+/CD8+ ratio were found in samples from patients with HPgV genotype 3 –high (p<0.01, +0.137) compared with HPgV –negative, and (p<0.05, +0.108) compared with –low (Fig 4E). The only significant effect of genotype 2 was seen in CD4+/CD8+ ratio: the mean ratio was significantly higher in genotype 2 –high as compared with HPgV- negative (p<0.05, +0.122) (Fig 4F).

To determine whether HIV infection time was a confounding factor and the specific effect of each HPgV condition, we sub-divided the ART-naïve population into recent (<6 months) or late (≥6 months) HIV infection subgroups (S3 Fig). We tested these subgroups by the Bonferroni test to compare the means of HIV markers within all possible combinations of HPgV viral load conditions and HIV subgroups. We found significantly lower means of HIV viral load in the HIV-late/HPgV-high (-0.85 HIV copies/ml log_10_) compared to HIV-late/HPgV-negative (p<0.05), and in the HIV-late/HPgV-high (-0.79 HIV copies/ml log_10_) compared to HIV-recent/HPgV-negative (p<0.05) (Table 2).

**Table 2:** Mean difference of HIV viral load in co-infected subjects according to time of HIV infection and HPgV viral load conditions.

***Conditions***	***HIV-recent HPgV-negative n = 29***	***HIV-late HPgV-negative n = 118***	***HIV-recent HPgV-low n = 37***	***HIV-late HPgV-low n = 82***	***HIV-recent HPgV-high n = 15***
***HIV-late HPgV-negative n = 118***	+0.062016				
***HIV-recent HPgV-low n = 37***	-0.429253	-0.491269			
***HIV-late HPgV-low n = 82***	-0.212959	-0.274974	+0.216294		
***HIV-recent HPgV-high n = 15***	-0.455913	-0.517929	-0.02666	-0.242955	
***HIV-late HPgV-high n = 22***	-0.795124*	-0.85714*	-0.365871	-0.582166	-0.339211

The table shows the differences of HIV viral load means between the 6 formed conditions (Bonferroni comparison test). HIV viral loads are expressed as genomic equivalents (GE)/ml (log_10_).

* = (p<0.05).

We also found significantly lower means of CD4^+^ cell counts in the HIV-late/HPgV-negative (-228 CD4^+^/mm^3^) compared to HIV-recent/HPgV-negative (p<0.05), and in the HIV-late/HPgV-low (-268 CD4^+^/mm^3^) compared to HIV-recent/HPgV-low (p<0.05). However, CD4^+^ cell counts were not significantly lower in the HIV late/HPgV-high (-133 CD4^+^/mm^3^) compared to HIV-recent/HPgV-high (p = 1.0) (Table 3).

**Table 3:** Mean difference of CD4^+^ cell counts (CD4^+^/mm^3^) in co-infected subjects according to time of HIV infection and HPgV viral load conditions.

***Conditions***	***HIV-recent HPgV-negative n = 29***	***HIV-late HPgV-negative n = 118***	***HIV-recent HPgV-low n = 37***	***HIV-late HPgV-low n = 82***	***HIV-recent HPgV-high n = 15***
***HIV-late HPgV-negative n = 118***	-228.079*				
***HIV-recent HPgV-low n = 37***	+55.7465	+283.826 ^‡^			
***HIV-late HPgV-low n = 82***	-212.819^‡^	+15.2604	-268.565*		
***HIV-recent HPgV-high n = 15***	+55.1609	+283.24 ^‡^	-.585586	+267.98^‡^	
***HIV-late HPgV-high n = 22***	-78.6724	+149.407	-134.419	+134.146	**-133.833**

The Table shows the differences of CD4^+^ cell count means between the 6 formed conditions (Bonferroni comparison test). CD4^+^ cell counts are expressed as (CD4^+^/mm^3^). **Underlined** = not statistically significant but relevant to HIV progression.

* = (p<0.05).

^**‡**^ = statistically significant although not necessarily related to HPgV infection.

In addition, there was a significantly higher CD4^+^/CD8^+^ ratio in the HIV-late/HPgV-high (+0.17) compared to the HIV-late/HPgV-negative (p<0.05) (Table 4).

**Table 4:** Mean difference of CD4^+^/CD8^+^ ratio in co-infected subjects according to time of HIV infection and HPgV viral load condition.

***Conditions***	***HIV-recent HPgV-negative n = 29***	***HIV-late HPgV-negative n = 118***	***HIV-recent HPgV-low n = 37***	***HIV-late HPgV-low n = 82***	***HIV-recent HPgV-high n = 15***
***HIV-late HPgV-negative n = 118***	-0.056481				
***HIV-recent HPgV-low n = 37***	-0.101481	-0.045			
***HIV-late HPgV-low n = 82***	-0.017579	+0.038902	+0.083902		
***HIV-recent HPgV-high n = 15***	-0.086815	-0.030333	+0.014667	-0.069236	
***HIV-late HPgV-high n = 22***	+0.118064	+0.174545*	+0.219545^‡^	+0.135643	+0.204879

The Table shows the differences of CD4^+^/CD8^+^ ratio means between the 6 formed conditions (Bonferroni comparison test). CD4^+^/CD8^+^ ratio is expressed in natural numbers.

* = p<0.05.

^**‡**^ = statistically significant although not necessarily related to HPgV infection.

## Discussion

The clinical relevance of HPgV infection resides in its beneficial effect on HIV and HCV disease progression and decrease in Ebola virus mortality [17,35–37]. On the other hand, HPgV infection has been associated with the long-term development of non-Hodgkin’s lymphoma [1,2]. Although there is evidence that HPgV infection is associated with a delay in HIV disease progression in co-infected patients, the underlying mechanism of their interaction and relationship to the host remains unclear [18,38–40]. One of the aims of this study was to assess HPgV infection in Mexico. We found that the prevalence of HPgV viremia (2.9%) in healthy blood donors and (33%) in HIV patients was in the range of those reported from other countries [17,22,41–44]. The prevalent genotypes we found, 3 (58.6%) and 2 (33.7%), could be associated with our geographical location and population’s ethnicity, since these are the genotypes frequent in Europe and the United States [9,45]. Genotype 3 is frequent in Asia and has been associated with Amerindian ancestry [11,12,14,15].

At present, 93% of the Mexican population are mestizo, with a genetic heterogeneity conformed by an asymmetric admixture of three populations, i.e., Amerindians, European and Africans. The proportion of the genetic admixture varies from the country’s north to the south between Amerindian (38 to 75%), European (50 to 8.5%) and African (9.4 to 18.5%). The average genetic proportion in Mexico is 59% Amerindian, 33% European, and 13% African [46]. These proportions are similar to the prevalence of HPgV genotypes we found, suggesting a relationship of HPgV genotype with race. HPgV causes chronic infections, which could lead to co-infections or a recombination between different genotypes [24,47,48], especially in regions with a higher prevalence of more than one genotype. We found mixed infections with HPgV genotypes 2/3 in 30 of 445 (6.7%) genotyped samples. HPgV genotype co-infections should be investigated to determine the implications of multiple HPgV co-infections. In our study, we also attempted to define the viral load pattern in HPgV-infected individuals and co-infected patients. When we analysed the density distribution of viremia levels in healthy blood donors and co-infected ART-naïve patients by Finite Mixture Models (Fig 2A and 2B), we clearly identified two independent clusters with low or high viral loads in both populations, with cut-off points that were almost identical (5.06log_10_ for healthy blood donors and 5.07 log_10_ for co-infected ART-naïve patients). The composition of -low and high-viral load clusters appeared to be independent of HIV infection, suggesting a differential regulation of HPgV replication. Although we found a bimodal pattern in HPgV viral load in ART-experienced patients (S4 Fig), we excluded co-infected HAART patients from this study to avoid HIV antiviral therapy as a confounding factor, since previous reports have found an increase of HPgV viral load in HIV co-infected patients on HAART [49], [49]. This bimodal pattern could be the result of distinct viral subtypes, or could be due to host immunity or anti-viral regulation or both. Since we also found no significant differences in the proportion of genotypes 2 and 3 belonging to HPgV-low or -high conditions, our results suggest that the bimodal pattern of HPgV viral load is independent of HPgV genotype.

Thus, it is important to characterise the whole genome sequence of strains belonging to the -low and -high viral load clusters, and also to characterise the cellular immune response expression of patients in each cluster.

To understand co-infection, we evaluated the effects of HPgV viremia on HIV progression markers (Fig 3A–3C). We compared the means of surrogate markers of HIV progression (HIV viral load, CD4^+^ cell counts, and CD4^+^/CD8^+^ ratio) between HPgV viral load conditions (-negative, -low, and -high). HPgV-positive patients compared to HPgV-negative patients had significantly lower HIV loads and higher CD4+ counts and CD4+/CD8+ ratios. Furthermore, enhanced beneficial effects were found in the HPgV-high condition patients, in all comparisons. These effects were also observed when surrogate markers of HIV progression were analysed according to the most prevalent genotypes, 3 and 2, although the effect was stronger with genotype 3 than with genotype 2 (Fig 4A–4F). Although these beneficial effects of HPgV co-infection with HIV were independent of HPgV genotype, our results suggest that genotypes could have different mechanisms for inducing such effects.

It is widely known that the progression of HIV disease is directly related with the time of infection, with late HIV infections associated with CD4^+^ cell depletion and increased HIV replication [50]. To determine whether the time of HIV infection was a confounding factor in our study, we analysed the effect of HPgV viral load conditions according to recent or late HIV infection (S1 Table). Using a multiple comparative analysis, we observed that in late HIV infection, high HPgV viremia was associated with a significant reduction of HIV viral load and significantly higher CD4^+^ counts (Tables 2 and 3). We also found that in late HIV infection, undetectable or low levels of HPgV viremia were associated with a significant decrease in CD4+ cell counts; in contrast, for high HPgV viremia in late HIV infection, the decrease in CD4+ count was not significant, supporting the protective effect of HPgV replication on CD4+cell counts, as suggested by others [38]. These results could suggest a correlation between high HPgV viral loads and improved beneficial effects in co-infected patients regardless of HIV infection time.

Our results support other studies, which suggest that HPgV and HIV interfere with each other for replication, independent of HIV infection time [40,51–53]. Moreover, our findings suggest that HPgV plays a major role in this interaction. In this study, we found that a minimum threshold of HPgV viral load is required to induce a beneficial effect on some markers of HIV progression.

The strength of the study is the relatively large sample of healthy blood donors and HIV ART-naïve patients analysed as well as the major prevalence of two genotypes, 2 and 3, allowing us to compare the behaviour of each one. A limitation of this study is its cross-sectional design; a longitudinal design with multiple samples taken during a long follow-up period would be ideal.

The effects of HPgV infection in humans are not completely characterised, and more in-depth studies are needed to elucidate the underlying mechanisms of the beneficial effects described in co-infected patients and the factors that determine the differences in HPgV viral load levels. To continue this path, we are working on expression assays to understand the changes that occur in the host’s immune activation and antiviral regulation during HPgV infection and HPgV/HIV co-infection. Another issue that should be explored is whether genome characteristics or host condition defines the two HPgV levels of replication (low and high).

The field of HPgV research must be encouraged, since the beneficial effects of HPgV may not be limited to HIV infection. Consistent with this, studies analyzing HPgV co-infection have shown a slower progression of liver fibrosis in Hepatitis C virus co-infected patients [36,37], and improved survival among those infected with Ebola virus [35]. Our study proposes that the viral load cut-off point should be considered for future HPgV co-infection studies.

## Supporting information

S1 Table Data reporting of HIV-positive ART-naïve patient. The Table shows each HIV-positive ART-naïve patient’s ID, HIV and HPgV viral loads, CD4^+^ cell counts, CD4^+^/CD8^+^ ratio, HPgV viral load condition, and HIV infection time. N/D means not determined. Viral loads are reported in Genome Equivalents (GE)/ml. Patient ID is a consecutive number given for identification in this study and is not linked to any patient’s Hospital files.
(PDF)

S1 Fig Flowchart of samples analysed for HPgV. The flowchart shows the subset division of the samples used for HPgV viremia, viral load and genotype determination.
(TIF)

S2 Fig Flowchart of samples included in the Finite Mixture Models analysis. FMM analysis of HPgV viral load heterogeneity in healthy blood donors and co-infected ART-naïve patients. The co-infected HIV patients in HAART were not included in this analysis.
(TIF)

S3 Fig Flowchart of samples analysed to determine the effect of HPgV viral load on surrogate markers of HIV progression. The flowchart shows the subsets division of the samples according to HPgV conditions and HIV infection time.
(TIF)

S4 Fig Two-component cluster mixture model fit for weighted HPgV viral load from co-infected ART-experienced patients. Density distribution (bars) of HPgV viral load (log10 genome equivalents) from co-infected ART-experienced patients. Gaussian curves indicate the fitted low (red line), high (green line), mean component curves (μ), the number of patients (n) and proportion (λ) in each population component. The clusters’ cut-off point is the intersection of the curves.
(TIF)

S5 Fig Genetic diversity of mixed HPgV infections. The phylogenetic analysis was constructed from partial nucleotide sequences of 5’UTR region by using the neighbor-joining method with MEGA software version 6. Reference strains were selected from GenBank and included. Bootstrap values are percentages of 1000 iterations. Reference strains were labelled as follows: GenBank accession number + word “GEN” + corresponding HPgV genotype, e.g. AB003289 GEN 2a. Patients samples are identified by Patient ID* for genotypes 2 and Patient ID for genotypes 3.
(PDF)
