# Supplementary material for: High HPgV replication is associated with improved surrogate markers of HIV progression
Source: PLoS One. 2017 Sep 14;12(9):e0184494. doi: 10.1371/journal.pone.0184494 (PMC5598987; doi:10.1371/journal.pone.0184494)
Supplement: S1 Table — The Table shows each HIV-positive ART-naïve patient’s ID, HIV and HPgV viral loads, CD4+ cell counts, CD4+/CD8+ ratio, HPgV viral load condition, and HIV infection time. N/D means not determined. Viral loads are reported in Genome Equivalents (GE)/ml. Patient ID is a consecutive number given for identification in this study and is not linked to any patient’s Hospital files. (PDF) [file pone.0184494.s001.pdf]

Patient ID	HIV GE/ml (log10)	CD4+ cell counts	HPgV GE/ml (log10)	HPgV condition	CD4+/CD8+ ratio	HIV Infection Time	HPgV Genotype	Age in years	Gender	Region	Civil Status	Scholarship	Employment	Risk Factor
1	6	100	NA	Negative	0.11	ND	NA	32	M	Jalisco	Unknown	Unknown	Unknown	Unknown
2	5.57	30	NA	Negative	0.06	ND	NA	41	M	Jalisco	Unknown	Unknown	Unknown	Unknown
3	4.60	458	NA	Negative	0.98	ND	NA	30	F	Jalisco	Unknown	Unknown	Unknown	Unknown
4	5.30	49	NA	Negative	0.04	ND	NA	41	M	Jalisco	Unknown	Unknown	Unknown	Unknown
5	7.00	343	NA	Negative	1.06	ND	NA	21	M	Jalisco	Unknown	Unknown	Unknown	Unknown
6	5.01	148	NA	Negative	ND	ND	NA	28	M	Jalisco	Unknown	Unknown	Unknown	Unknown
7	5.34	192	NA	Negative	ND	ND	NA	28	F	Jalisco	Unknown	Unknown	Unknown	Unknown
8	5.13	185	NA	Negative	0.22	ND	NA	26	M	Jalisco	Unknown	Unknown	Unknown	Unknown
9	5.20	30	NA	Negative	0.11	ND	NA	23	M	Jalisco	Unknown	Unknown	Unknown	Unknown
10	3.69	232	NA	Negative	0.17	ND	NA	31	F	Guerrero	Unknown	Unknown	Unknown	Unknown
11	3.82	227	NA	Negative	0.26	ND	NA	40	F	Chiapas	Unknown	Unknown	Unknown	Unknown
12	5.37	257	NA	Negative	0.13	ND	NA	32	M	Jalisco	Unknown	Unknown	Unknown	Unknown
13	5.49	235	NA	Negative	0.19	ND	NA	39	M	DF	Unknown	Unknown	Unknown	Unknown
14	5.16	149	NA	Negative	0.26	ND	NA	41	M	Jalisco	Unknown	Unknown	Unknown	Unknown
15	5.43	52	NA	Negative	0.04	ND	NA	36	M	Jalisco	Unknown	Unknown	Unknown	Unknown
16	3.05	48	NA	Negative	0.23	ND	NA	26	F	Jalisco	Unknown	Unknown	Unknown	Unknown
17	5.22	6	NA	Negative	0.01	ND	NA	28	M	Jalisco	Unknown	Unknown	Unknown	Unknown
18	5.13	293	NA	Negative	0.2	ND	NA	40	F	Jalisco	Unknown	Unknown	Unknown	Unknown
19	5.54	99	NA	Negative	0.27	ND	NA	30	M	Jalisco	Unknown	Unknown	Unknown	Unknown
20	4.47	105	NA	Negative	0.21	ND	NA	42	M	DF	Unknown	Unknown	Unknown	Unknown
21	5.60	21	NA	Negative	0.14	ND	NA	51	M	DF	Unknown	Unknown	Unknown	Unknown
22	4.70	221	NA	Negative	0.23	ND	NA	Unknown	M	Edo. Méx	Unknown	Unknown	Unknown	Unknown
23	6.00	334	NA	Negative	0.23	ND	NA	26	M	DF	Unknown	Unknown	Unknown	Unknown
24	4.59	229	NA	Negative	0.47	ND	NA	23	M	Edo. Méx	Unknown	Unknown	Unknown	Unknown
25	4.10	257	NA	Negative	0.35	ND	NA	26	F	Edo. Méx	Unknown	Unknown	Unknown	Unknown
26	5.68	68	NA	Negative	0.14	ND	NA	38	F	Jalisco	Unknown	Unknown	Unknown	Unknown
27	2.83	446	NA	Negative	0.49	ND	NA	24	F	Jalisco	Unknown	Unknown	Unknown	Unknown
28	4.66	431	NA	Negative	0.34	ND	NA	31	M	Edo. Méx	Unknown	Unknown	Unknown	Unknown
29	4.38	373	NA	Negative	0.93	ND	NA	40	M	Edo. Méx	Unknown	Unknown	Unknown	Unknown
30	5.77	51	NA	Negative	0.08	ND	NA	28	M	DF	Unknown	Unknown	Unknown	Unknown

31	5.19	244	NA	Negative	0.14	ND	NA	38	M	DF	Unknown	Unknown	Unknown	Unknown
32	4.85	6	NA	Negative	0.02	ND	NA	Unknown	M	Unknown	Unknown	Unknown	Unknown	Unknown
33	5.34	97	NA	Negative	0.04	ND	NA	42	M	DF	Unknown	Unknown	Unknown	Unknown
34	3.99	396	NA	Negative	0.47	ND	NA	33	F	DF	Unknown	Unknown	Unknown	Unknown
35	6.00	112	NA	Negative	0.05	ND	NA	43	M	Jalisco	Unknown	Unknown	Unknown	Unknown
36	6.00	3	NA	Negative	0.02	ND	NA	32	M	Jalisco	Unknown	Unknown	Unknown	Unknown
37	4.52	457	NA	Negative	0.47	ND	NA	38	M	Tijuana	Unknown	Unknown	Unknown	Unknown
38	4.03	336	NA	Negative	0.26	ND	NA	28	M	Edo. Méx	Unknown	Unknown	Unknown	Unknown
39	4.93	54	NA	Negative	0.05	ND	NA	36	M	Veracruz	Unknown	Unknown	Unknown	Unknown
40	4.06	330	NA	Negative	0.36	ND	NA	19	M	DF	Unknown	Unknown	Unknown	Unknown
41	5.30	72	NA	Negative	0.17	ND	NA	26	M	Jalisco	Unknown	Unknown	Unknown	Unknown
42	5.54	285	NA	Negative	0.31	ND	NA	42	M	Jalisco	Unknown	Unknown	Unknown	Unknown
43	5.30	22	NA	Negative	0.03	ND	NA	45	M	Jalisco	Unknown	Unknown	Unknown	Unknown
44	4.54	295	NA	Negative	0.25	ND	NA	39	M	Veracruz	Unknown	Unknown	Unknown	Unknown
45	5.57	68	NA	Negative	0.06	ND	NA	44	M	Edo. Méx	Unknown	Unknown	Unknown	Unknown
46	4.27	25	NA	Negative	0.05	ND	NA	45	F	Veracruz	Unknown	Unknown	Unknown	Unknown
47	4.59	463	NA	Negative	0.3	ND	NA	30	M	Veracruz	Unknown	Unknown	Unknown	Unknown
48	5.87	14	NA	Negative	0.01	ND	NA	29	M	Veracruz	Unknown	Unknown	Unknown	Unknown
49	5.39	478	NA	Negative	0.25	ND	NA	27	M	Veracruz	Unknown	Unknown	Unknown	Unknown
50	4.79	171	NA	Negative	0.15	ND	NA	33	M	DF	Unknown	Unknown	Unknown	Unknown
51	5.23	189	NA	Negative	0.18	ND	NA	30	M	Veracruz	Unknown	Unknown	Unknown	Unknown
52	4.68	241	NA	Negative	0.68	ND	NA	20	F	Jalisco	Unknown	Unknown	Unknown	Unknown
53	4.16	339	NA	Negative	0.58	ND	NA	37	F	Jalisco	Unknown	Unknown	Unknown	Unknown
54	4.65	36	NA	Negative	0.03	ND	NA	64	M	Jalisco	Unknown	Unknown	Unknown	Unknown
55	4.56	636	NA	Negative	0.25	ND	NA	27	M	Edo. Méx	Unknown	Unknown	Unknown	Unknown
56	5.02	19	NA	Negative	0.1	ND	NA	25	F	Edo. Méx	Unknown	Unknown	Unknown	Unknown
57	4.09	373	NA	Negative	0.51	ND	NA	27	F	Edo. Méx	Unknown	Unknown	Unknown	Unknown
58	4.72	3	NA	Negative	0.03	ND	NA	28	M	Jalisco	Unknown	Unknown	Unknown	Unknown
59	5.16	270	NA	Negative	0.2	ND	NA	30	M	Edo. Méx	Unknown	Unknown	Unknown	Unknown
60	5.13	283	NA	Negative	0.14	ND	NA	34	M	DF	Unknown	Unknown	Unknown	Unknown
61	3.65	194	NA	Negative	0.21	ND	NA	25	F	Edo. Méx	Unknown	Unknown	Unknown	Unknown
62	5.97	5	NA	Negative	0.02	ND	NA	24	M	Edo. Méx	Unknown	Unknown	Unknown	Unknown
63	5.19	102	NA	Negative	0.08	ND	NA	41	M	Veracruz	Unknown	Unknown	Unknown	Unknown

64	5.17	324	NA	Negative	0.1	ND	NA	6	M	Veracruz	Unknown	Unknown	Unknown	Unknown
65	5.51	27	NA	Negative	0.05	ND	NA	24	M	DF	Unknown	Unknown	Unknown	Unknown
66	4.88	245	NA	Negative	0.33	ND	NA	34	M	Jalisco	Unknown	Unknown	Unknown	Unknown
67	5.98	105	NA	Negative	0.09	ND	NA	38	M	DF	Unknown	Unknown	Unknown	Unknown
68	4.89	474	NA	Negative	0.57	ND	NA	22	M	Edo. Méx	Unknown	Unknown	Unknown	Unknown
69	6.00	48	NA	Negative	0.12	ND	NA	41	F	Jalisco	Unknown	Unknown	Unknown	Unknown
70	6.00	93	NA	Negative	0.1	ND	NA	40	M	Edo. Méx	Unknown	Unknown	Unknown	Unknown
71	6.00	58	NA	Negative	0.13	ND	NA	31	M	Morelos	Unknown	Unknown	Unknown	Unknown
72	4.15	242	NA	Negative	0.1	ND	NA	19	M	Jalisco	Unknown	Unknown	Unknown	Unknown
73	5.15	87	NA	Negative	0.15	ND	NA	29	M	DF	Unknown	Unknown	Unknown	Unknown
74	4.37	481	NA	Negative	0.66	ND	NA	24	F	Morelos	Unknown	Unknown	Unknown	Unknown
75	4.83	224	NA	Negative	0.34	ND	NA	30	M	DF	Unknown	Unknown	Unknown	Unknown
76	5.28	209	NA	Negative	0.2	ND	NA	28	F	DF	Unknown	Unknown	Unknown	Unknown
77	6.26	20	NA	Negative	0.05	ND	NA	26	M	Jalisco	Unknown	Unknown	Unknown	Unknown
78	6.08	26	NA	Negative	0.13	ND	NA	42	M	Jalisco	Unknown	Unknown	Unknown	Unknown
79	5.57	128	NA	Negative	0.24	ND	NA	39	F	Morelos	Unknown	Unknown	Unknown	Unknown
80	6.36	146	NA	Negative	0.15	ND	NA	28	M	Jalisco	Unknown	Unknown	Unknown	Unknown
81	5.00	17	NA	Negative	0.04	ND	NA	42	M	Jalisco	Unknown	Unknown	Unknown	Unknown
82	5.23	376	NA	Negative	0.12	ND	NA	16	M	Morelos	Unknown	Unknown	Unknown	Unknown
83	5.43	185	NA	Negative	0.11	ND	NA	35	M	Morelos	Unknown	Unknown	Unknown	Unknown
84	5.33	67	NA	Negative	0.1	ND	NA	47	F	Jalisco	Unknown	Unknown	Unknown	Unknown
85	4.65	337	NA	Negative	0.25	ND	NA	22	M	DF	Unknown	Unknown	Unknown	Unknown
86	6.00	105	NA	Negative	0.19	ND	NA	50	M	Morelos	Unknown	Unknown	Unknown	Unknown
87	3.56	454	NA	Negative	0.66	ND	NA	23	M	DF	Unknown	Unknown	Unknown	Unknown
88	5.45	27	NA	Negative	0.04	ND	NA	36	M	Jalisco	Unknown	Unknown	Unknown	Unknown
89	5.57	77	NA	Negative	0.11	ND	NA	27	M	Michoacán	Unknown	Unknown	Unknown	Unknown
90	5.67	218	NA	Negative	0.22	ND	NA	31	M	Jalisco	Unknown	Unknown	Unknown	Unknown
91	3.57	365	NA	Negative	0.45	ND	NA	33	M	Veracruz	Unknown	Unknown	Unknown	Unknown
92	6.00	94	NA	Negative	0.09	ND	NA	35	M	DF	Unknown	Unknown	Unknown	Unknown
93	3.94	335	NA	Negative	0.51	ND	NA	30	M	Edo. Méx	Unknown	Unknown	Unknown	Unknown
94	5.02	61	NA	Negative	0.12	ND	NA	42	F	Tijuana	Unknown	Unknown	Unknown	Unknown
95	4.71	215	NA	Negative	0.22	ND	NA	27	M	Edo. Méx	Unknown	Unknown	Unknown	Unknown
96	3.99	323	NA	Negative	0.62	ND	NA	29	M	Morelos	Unknown	Unknown	Unknown	Unknown

97	4.91	380	NA	Negative	0.2	ND	NA	38	M	Morelos	Unknown	Unknown	Unknown	Unknown
98	5.68	5	NA	Negative	0.03	ND	NA	45	M	Jalisco	Unknown	Unknown	Unknown	Unknown
99	4.39	131	NA	Negative	0.22	ND	NA	30	M	Jalisco	Unknown	Unknown	Unknown	Unknown
100	5.91	172	NA	Negative	0.33	ND	NA	23	M	Edo. Méx	Unknown	Unknown	Unknown	Unknown
101	4.49	424	NA	Negative	0.25	ND	NA	30	M	DF	Unknown	Unknown	Unknown	Unknown
102	5.36	246	NA	Negative	0.17	ND	NA	24	M	Edo. Méx	Unknown	Unknown	Unknown	Unknown
103	4.90	332	NA	Negative	0.22	ND	NA	26	M	DF	Unknown	Unknown	Unknown	Unknown
104	4.37	347	NA	Negative	0.36	ND	NA	37	F	DF	Unknown	Unknown	Unknown	Unknown
105	4.82	516	NA	Negative	0.34	ND	NA	44	F	Edo. Méx	Unknown	Unknown	Unknown	Unknown
106	5.55	9	NA	Negative	0.04	ND	NA	24	M	Edo. Méx	Unknown	Unknown	Unknown	Unknown
107	5.08	257	NA	Negative	0.28	ND	NA	42	M	Jalisco	Unknown	Unknown	Unknown	Unknown
108	5.03	117	NA	Negative	0.19	ND	NA	34	M	Jalisco	Unknown	Unknown	Unknown	Unknown
109	6.07	337	NA	Negative	0.7	ND	NA	7 months	F	Veracruz	Unknown	Unknown	Unknown	Unknown
110	5.00	87	NA	Negative	0.17	ND	NA	28	M	Veracruz	Unknown	Unknown	Unknown	Unknown
111	5.20	127	NA	Negative	0.08	ND	NA	58	M	Morelos	Unknown	Unknown	Unknown	Unknown
112	4.04	288	NA	Negative	0.63	ND	NA	30	F	Edo. Méx	Unknown	Unknown	Unknown	Unknown
113	5.03	125	NA	Negative	0.09	ND	NA	52	M	Jalisco	Unknown	Unknown	Unknown	Unknown
114	5.95	194	NA	Negative	0.31	ND	NA	29	M	Jalisco	Unknown	Unknown	Unknown	Unknown
115	5.22	70	NA	Negative	0.06	ND	NA	32	M	Edo. Méx	Unknown	Unknown	Unknown	Unknown
116	5.69	105	NA	Negative	0.11	ND	NA	35	M	Jalisco	Unknown	Unknown	Unknown	Unknown
117	5.99	75	NA	Negative	0.11	ND	NA	36	F	Edo. Méx	Unknown	Unknown	Unknown	Unknown
118	4.03	227	NA	Negative	0.38	ND	NA	56	M	Edo. Méx	Unknown	Unknown	Unknown	Unknown
119	5.61	44	NA	Negative	0.06	ND	NA	36	M	Edo. Méx	Unknown	Unknown	Unknown	Unknown
120	4.08	282	NA	Negative	0.23	ND	NA	35	M	DF	Unknown	Unknown	Unknown	Unknown
121	4.39	49	NA	Negative	0.15	ND	NA	36	M	Veracruz	Unknown	Unknown	Unknown	Unknown
122	3.38	383	NA	Negative	0.84	ND	NA	30	F	Morelos	Unknown	Unknown	Unknown	Unknown
123	4.59	54	NA	Negative	0.22	ND	NA	27	M	Morelos	Unknown	Unknown	Unknown	Unknown
124	3.84	244	NA	Negative	0.33	ND	NA	22	M	Morelos	Unknown	Unknown	Unknown	Unknown
125	4.75	89	NA	Negative	0.09	ND	NA	32	M	Jalisco	Unknown	Unknown	Unknown	Unknown
126	4.28	125	NA	Negative	0.16	ND	NA	30	M	Jalisco	Unknown	Unknown	Unknown	Unknown
127	5.67	9	NA	Negative	0.15	ND	NA	26	M	Veracruz	Unknown	Unknown	Unknown	Unknown
128	5.36	25	NA	Negative	0.02	ND	NA	40	M	Edo. Méx	Unknown	Unknown	Unknown	Unknown
129	5.01	238	NA	Negative	0.2	ND	NA	23	M	Veracruz	Unknown	Unknown	Unknown	Unknown

130	4.76	17	NA	Negative	0.09	ND	NA	42	M	Morelos	Unknown	Unknown	Unknown	Unknown
131	5.66	5	NA	Negative	0.07	ND	NA	35	M	Edo. Méx	Unknown	Unknown	Unknown	Unknown
132	5.36	13	NA	Negative	0.06	ND	NA	30	M	Jalisco	Unknown	Unknown	Unknown	Unknown
133	4.91	158	NA	Negative	0.18	ND	NA	43	F	Jalisco	Unknown	Unknown	Unknown	Unknown
134	3.87	211	NA	Negative	0.42	ND	NA	Unknown	F	Jalisco	Unknown	Unknown	Unknown	Unknown
135	5.27	219	NA	Negative	0.21	ND	NA	21	M	Jalisco	Unknown	Unknown	Unknown	Unknown
136	4.85	198	NA	Negative	0.09	ND	NA	48	M	Jalisco	Unknown	Unknown	Unknown	Unknown
137	5.49	4	NA	Negative	0.03	ND	NA	28	M	Veracruz	Unknown	Unknown	Unknown	Unknown
138	5.25	47	NA	Negative	0.05	ND	NA	35	M	Veracruz	Unknown	Unknown	Unknown	Unknown
139	3.60	590	NA	Negative	0.69	ND	NA	36	F	Edo. Méx	Unknown	Unknown	Unknown	Unknown
140	4.83	145	NA	Negative	0.08	ND	NA	40	M	Edo. Méx	Unknown	Unknown	Unknown	Unknown
141	4.55	134	NA	Negative	0.12	ND	NA	37	F	Edo. Méx	Unknown	Unknown	Unknown	Unknown
142	5.10	189	NA	Negative	0.39	ND	NA	24	M	DF	Unknown	Unknown	Unknown	Unknown
143	4.00	243	NA	Negative	0.6	ND	NA	28	M	Morelos	Unknown	Unknown	Unknown	Unknown
144	4.62	400	NA	Negative	0.63	ND	NA	26	F	Morelos	Unknown	Unknown	Unknown	Unknown
145	4.67	248	NA	Negative	0.23	ND	NA	36	M	Veracruz	Unknown	Unknown	Unknown	Unknown
146	4.94	404	NA	Negative	0.39	ND	NA	22	M	Morelos	Unknown	Unknown	Unknown	Unknown
147	5.81	76	NA	Negative	0.15	ND	NA	26	M	DF	Unknown	Unknown	Unknown	Unknown
148	4.77	342	NA	Negative	0.38	ND	NA	25	M	Veracruz	Unknown	Unknown	Unknown	Unknown
149	5.56	132	NA	Negative	0.28	ND	NA	39	M	Jalisco	Unknown	Unknown	Unknown	Unknown
150	3.36	88	NA	Negative	0.12	ND	NA	39	M	Jalisco	Unknown	Unknown	Unknown	Unknown
151	5.37	90	NA	Negative	0.1	ND	NA	28	M	Jalisco	Unknown	Unknown	Unknown	Unknown
152	5.68	117	NA	Negative	0.2	ND	NA	30	M	Jalisco	Unknown	Unknown	Unknown	Unknown
153	4.91	50	NA	Negative	0.11	ND	NA	39	F	Jalisco	Unknown	Unknown	Unknown	Unknown
154	4.58	258	NA	Negative	0.22	ND	NA	30	M	Edo. Méx	Unknown	Unknown	Unknown	Unknown
155	4.68	459	NA	Negative	0.28	ND	NA	51	F	Veracruz	Unknown	Unknown	Unknown	Unknown
156	4.11	104	NA	Negative	0.17	ND	NA	48	M	Veracruz	Unknown	Unknown	Unknown	Unknown
157	4.78	599	NA	Negative	0.51	ND	NA	30	M	Veracruz	Unknown	Unknown	Unknown	Unknown
158	4.13	304	NA	Negative	0.31	ND	NA	59	M	Veracruz	Unknown	Unknown	Unknown	Unknown
159	4.91	482	NA	Negative	0.26	ND	NA	58	M	Veracruz	Unknown	Unknown	Unknown	Unknown
160	4.95	224	NA	Negative	0.14	ND	NA	26	M	DF	Unknown	Unknown	Unknown	Unknown
161	6.23	38	NA	Negative	0.07	ND	NA	35	M	Morelos	Unknown	Unknown	Unknown	Unknown
162	4.23	64	NA	Negative	0.06	ND	NA	54	M	Jalisco	Unknown	Unknown	Unknown	Unknown

163	4.87	325	NA	Negative	0.42	ND	NA	46	M	Jalisco	Unknown	Unknown	Unknown	Unknown
164	5.39	21	NA	Negative	0.04	ND	NA	23	M	Edo. Méx	Unknown	Unknown	Unknown	Unknown
165	5.50	73	NA	Negative	0.08	ND	NA	37	M	Veracruz	Unknown	Unknown	Unknown	Unknown
166	5.54	192	NA	Negative	0.19	ND	NA	36	M	Veracruz	Unknown	Unknown	Unknown	Unknown
167	4.96	137	NA	Negative	0.26	ND	NA	34	M	Edo. Méx	Unknown	Unknown	Unknown	Unknown
168	4.88	268	NA	Negative	0.13	ND	NA	75	M	Morelos	Unknown	Unknown	Unknown	Unknown
169	4.98	282	NA	Negative	0.38	ND	NA	27	M	Morelos	Unknown	Unknown	Unknown	Unknown
170	3.85	209	NA	Negative	0.37	ND	NA	74	M	Veracruz	Unknown	Unknown	Unknown	Unknown
171	4.91	18	NA	Negative	0.04	ND	NA	25	M	DF	Unknown	Unknown	Unknown	Unknown
172	5.40	29	NA	Negative	0.1	ND	NA	26	M	Edo. Méx	Unknown	Unknown	Unknown	Unknown
173	5.31	626	NA	Negative	0.39	ND	NA	24	M	DF	Unknown	Unknown	Unknown	Unknown
174	5.38	281	NA	Negative	0.26	ND	NA	41	M	Edo. Méx	Unknown	Unknown	Unknown	Unknown
175	3.84	146	NA	Negative	0.14	ND	NA	41	F	Edo. Méx	Unknown	Unknown	Unknown	Unknown
176	5.79	31	NA	Negative	0.08	ND	NA	36	F	Morelos	Unknown	Unknown	Unknown	Unknown
177	5.21	19	NA	Negative	0.04	ND	NA	30	M	Unknown	Unknown	Unknown	Unknown	Unknown
178	3.27	470	NA	Negative	0.46	ND	NA	Unknown	M	DF	Unknown	Unknown	Unknown	Unknown
179	4.54	100	NA	Negative	0.2	ND	NA	30	F	Edo. Méx	Unknown	Unknown	Unknown	Unknown
180	5.44	172	NA	Negative	0.21	ND	NA	32	M	Edo. Méx	Unknown	Unknown	Unknown	Unknown
181	4.49	255	NA	Negative	0.42	ND	NA	30	F	Edo. Méx	Unknown	Unknown	Unknown	Unknown
182	4.58	366	NA	Negative	0.47	ND	NA	31	M	Nuevo León	Unknown	Unknown	Unknown	Unknown
183	4.29	106	NA	Negative	0.19	ND	NA	27	M	Jalisco	Unknown	Unknown	Unknown	Unknown
184	4.78	401	NA	Negative	0.47	ND	NA	27	M	Jalisco	Unknown	Unknown	Unknown	Unknown
185	5.51	14	NA	Negative	0.16	ND	NA	54	M	Jalisco	Unknown	Unknown	Unknown	Unknown
186	5.08	48	NA	Negative	0.11	ND	NA	45	M	DF	Unknown	Unknown	Unknown	Unknown
187	4.13	354	NA	Negative	0.52	ND	NA	35	F	Nuevo León	Unknown	Unknown	Unknown	Unknown
188	4.75	79	NA	Negative	0.09	ND	NA	40	M	Nuevo León	Unknown	Unknown	Unknown	Unknown
189	5.71	388	NA	Negative	0.18	ND	NA	23	M	Veracruz	Unknown	Unknown	Unknown	Unknown
190	5.72	57	NA	Negative	0.08	ND	NA	37	M	Jalisco	Unknown	Unknown	Unknown	Unknown
191	5.72	73	NA	Negative	0.08	ND	NA	28	M	Edo. Méx	Unknown	Unknown	Unknown	Unknown
192	4.33	297	NA	Negative	0.28	ND	NA	34	M	Edo. Méx	Unknown	Unknown	Unknown	Unknown
193	5.73	106	NA	Negative	0.08	ND	NA	32	F	Edo. Méx	Unknown	Unknown	Unknown	Unknown
194	5.50	22	NA	Negative	0.15	ND	NA	26	M	Edo. Méx	Unknown	Unknown	Unknown	Unknown

195	4.47	589	NA	Negative	0.81	ND	NA	21	F	Edo. Méx	Unknown	Unknown	Unknown	Unknown
196	5.32	77	NA	Negative	0.2	ND	NA	29	M	DF	Unknown	Unknown	Unknown	Unknown
197	4.52	286	NA	Negative	0.14	ND	NA	43	M	Morelos	Unknown	Unknown	Unknown	Unknown
198	5.11	24	NA	Negative	0.16	ND	NA	30	M	Veracruz	Unknown	Unknown	Unknown	Unknown
199	5.58	78	NA	Negative	0.13	ND	NA	28	M	Edo. Méx	Unknown	Unknown	Unknown	Unknown
200	4.27	98	NA	Negative	0.29	ND	NA	50	F	DF	Unknown	Unknown	Unknown	Unknown
201	6.29	43	NA	Negative	0.08	ND	NA	68	M	Morelos	Unknown	Unknown	Unknown	Unknown
202	4.04	376	NA	Negative	0.81	ND	NA	30	M	Edo. Méx	Unknown	Unknown	Unknown	Unknown
203	4.91	101	NA	Negative	0.41	ND	NA	27	M	Edo. Méx	Unknown	Unknown	Unknown	Unknown
204	5.56	287	NA	Negative	0.43	ND	NA	18	M	Edo. Méx	Unknown	Unknown	Unknown	Unknown
205	4.89	49	NA	Negative	0.13	ND	NA	42	M	DF	Unknown	Unknown	Unknown	Unknown
206	5.04	28	NA	Negative	0.2	ND	NA	34	F	Edo. Méx	Unknown	Unknown	Unknown	Unknown
207	4.12	318	NA	Negative	1.07	ND	NA	32	M	DF	Unknown	Unknown	Unknown	Unknown
208	5.39	68	NA	Negative	0.04	ND	NA	40	M	Edo. Méx	Unknown	Unknown	Unknown	Unknown
209	4.66	455	NA	Negative	0.63	ND	NA	30	F	Edo. Méx	Unknown	Unknown	Unknown	Unknown
210	4.42	271	NA	Negative	0.56	ND	NA	34	F	Edo. Méx	Unknown	Unknown	Unknown	Unknown
211	4.49	285	NA	Negative	0.2	ND	NA	29	M	Edo. Méx	Unknown	Unknown	Unknown	Unknown
212	5.09	270	NA	Negative	0.32	ND	NA	23	M	Morelos	Unknown	Unknown	Unknown	Unknown
213	4.26	233	NA	Negative	0.67	ND	NA	24	F	Morelos	Unknown	Unknown	Unknown	Unknown
214	4.47	190	NA	Negative	0.21	ND	NA	34	F	Morelos	Unknown	Unknown	Unknown	Unknown
215	5.74	167	NA	Negative	0.05	ND	NA	32	M	Morelos	Unknown	Unknown	Unknown	Unknown
216	4.12	36	NA	Negative	0.04	ND	NA	34	M	Jalisco	Unknown	Unknown	Unknown	Unknown
217	4.75	69	NA	Negative	0.08	ND	NA	27	M	Veracruz	Unknown	Unknown	Unknown	Unknown
218	5.23	59	NA	Negative	0.15	ND	NA	64	F	Jalisco	Unknown	Unknown	Unknown	Unknown
219	3.80	146	NA	Negative	0.58	ND	NA	29	F	Nuevo León	Unknown	Unknown	Unknown	Unknown
220	4.34	58	NA	Negative	0.11	ND	NA	37	F	Oaxaca	Unknown	Unknown	Unknown	Unknown
221	5.02	379	NA	Negative	0.59	ND	NA	22	M	Oaxaca	Unknown	Unknown	Unknown	Unknown
222	5.06	276	NA	Negative	0.18	ND	NA	24	M	Oaxaca	Unknown	Unknown	Unknown	Unknown
223	4.52	288	NA	Negative	0.32	ND	NA	32	M	Jalisco	Unknown	Unknown	Unknown	Unknown
224	5.43	66	NA	Negative	0.16	ND	NA	36	M	Morelos	Unknown	Unknown	Unknown	Unknown
225	5.49	44	NA	Negative	0.12	ND	NA	32	M	Morelos	Unknown	Unknown	Unknown	Unknown
226	4.58	241	NA	Negative	0.08	ND	NA	32	M	Edo. Méx	Unknown	Unknown	Unknown	Unknown
227	5.65	78	NA	Negative	0.1	ND	NA	33	M	DF	Unknown	Unknown	Unknown	Unknown

228	4.11	268	NA	Negative	0.29	ND	NA	51	M	DF	Unknown	Unknown	Unknown	Unknown
229	4.91	426	NA	Negative	0.61	ND	NA	24	M	Edo. Méx	Unknown	Unknown	Unknown	Unknown
230	5.98	150	NA	Negative	0.06	ND	NA	45	M	Edo. Méx	Unknown	Unknown	Unknown	Unknown
231	5.63	89	NA	Negative	0.08	ND	NA	41	M	DF	Unknown	Unknown	Unknown	Unknown
232	5.42	36	NA	Negative	0.12	ND	NA	28	M	Edo. Méx	Unknown	Unknown	Unknown	Unknown
233	5.55	105	NA	Negative	0.14	ND	NA	29	M	DF	Unknown	Unknown	Unknown	Unknown
234	4.48	257	NA	Negative	0.29	ND	NA	32	M	Jalisco	Unknown	Unknown	Unknown	Unknown
235	5.21	231	NA	Negative	0.2	ND	NA	30	M	Jalisco	Unknown	Unknown	Unknown	Unknown
236	5.89	23	NA	Negative	0.03	ND	NA	66	M	Veracruz	Unknown	Unknown	Unknown	Unknown
237	4.61	254	NA	Negative	0.14	ND	NA	29	M	Edo. Méx	Unknown	Unknown	Unknown	Unknown
238	4.53	94	NA	Negative	0.21	ND	NA	51	M	Morelos	Unknown	Unknown	Unknown	Unknown
239	5.57	81	NA	Negative	0.13	ND	NA	28	M	Veracruz	Unknown	Unknown	Unknown	Unknown
240	5.01	20	NA	Negative	0.17	ND	NA	40	M	Jalisco	Unknown	Unknown	Unknown	Unknown
241	3.83	40	NA	Negative	0.14	ND	NA	31	F	Jalisco	Unknown	Unknown	Unknown	Unknown
242	4.55	152	NA	Negative	0.17	ND	NA	36	M	Edo. Méx	Unknown	Unknown	Unknown	Unknown
243	4.66	486	NA	Negative	0.47	ND	NA	32	F	Edo. Méx	Unknown	Unknown	Unknown	Unknown
244	5.41	414	NA	Negative	0.25	ND	NA	51	M	Edo. Méx	Unknown	Unknown	Unknown	Unknown
245	4.35	86	NA	Negative	0.29	ND	NA	36	F	Morelos	Unknown	Unknown	Unknown	Unknown
246	5.48	47	NA	Negative	0.18	ND	NA	31	M	Morelos	Unknown	Unknown	Unknown	Unknown
247	4.81	342	NA	Negative	0.3	ND	NA	37	M	Morelos	Unknown	Unknown	Unknown	Unknown
248	4.22	121	NA	Negative	0.14	ND	NA	20	M	Edo. Méx	Unknown	Unknown	Unknown	Unknown
249	5.29	156	NA	Negative	0.34	ND	NA	44	M	DF	Unknown	Unknown	Unknown	Unknown
250	4.96	71	NA	Negative	0.09	ND	NA	42	M	Morelos	Unknown	Unknown	Unknown	Unknown
251	5.59	30	NA	Negative	0.08	ND	NA	27	M	Edo. Méx	Unknown	Unknown	Unknown	Unknown
252	4.98	175	NA	Negative	0.73	ND	NA	24	M	Jalisco	Unknown	Unknown	Unknown	Unknown
253	5.06	41	NA	Negative	0.18	ND	NA	44	M	Jalisco	Unknown	Unknown	Unknown	Unknown
254	4.81	313	NA	Negative	0.39	ND	NA	26	M	Jalisco	Unknown	Unknown	Unknown	Unknown
255	5.40	11	NA	Negative	0.05	ND	NA	47	M	Veracruz	Unknown	Unknown	Unknown	Unknown
256	4.65	394	NA	Negative	0.44	ND	NA	16	M	Edo. Méx	Unknown	Unknown	Unknown	Unknown
257	5.00	49	NA	Negative	0.13	ND	NA	36	F	Edo. Méx	Unknown	Unknown	Unknown	Unknown
258	5.26	116	NA	Negative	0.16	ND	NA	49	M	DF	Unknown	Unknown	Unknown	Unknown
259	4.58	265	NA	Negative	0.33	ND	NA	43	M	Morelos	Unknown	Unknown	Unknown	Unknown
260	5.83	107	NA	Negative	0.13	ND	NA	33	M	veracruz	Unknown	Unknown	Unknown	Unknown

261	4.93	68	NA	Negative	0.13	ND	NA	25	M	Edo. Méx	Unknown	Unknown	Unknown	Unknown
262	5.62	55	NA	Negative	0.12	ND	NA	36	M	Veracruz	Unknown	Unknown	Unknown	Unknown
263	5.75	396	NA	Negative	0.41	ND	NA	25	F	Jalisco	Unknown	Unknown	Unknown	Unknown
264	4.70	6	NA	Negative	0.02	ND	NA	27	M	Morelos	Unknown	Unknown	Unknown	Unknown
265	5.21	15	NA	Negative	0.02	ND	NA	34	M	Edo. Méx	Unknown	Unknown	Unknown	Unknown
266	4.51	67	NA	Negative	0.12	ND	NA	45	M	Edo. Méx	Unknown	Unknown	Unknown	Unknown
267	4.64	416	NA	Negative	0.68	ND	NA	48	M	Edo. Méx	Unknown	Unknown	Unknown	Unknown
268	5.78	48	NA	Negative	0.05	ND	NA	55	M	Edo. Méx	Unknown	Unknown	Unknown	Unknown
269	5.04	234	NA	Negative	0.27	ND	NA	47	M	DF	Unknown	Unknown	Unknown	Unknown
270	4.08	130	NA	Negative	0.13	ND	NA	51	M	Morelos	Unknown	Unknown	Unknown	Unknown
271	5.73	61	NA	Negative	0.19	ND	NA	37	M	Edo. Méx	Unknown	Unknown	Unknown	Unknown
272	4.36	670	NA	Negative	0.74	ND	NA	26	F	Morelos	Unknown	Unknown	Unknown	Unknown
273	3.73	1141	NA	Negative	0.77	ND	NA	16	M	Morelos	Unknown	Unknown	Unknown	Unknown
274	4.95	324	NA	Negative	0.31	ND	NA	56	M	Morelos	Unknown	Unknown	Unknown	Unknown
275	5.60	310	NA	Negative	0.17	ND	NA	40	M	DF	Unknown	Unknown	Unknown	Unknown
276	5.37	59	NA	Negative	0.22	ND	NA	54	M	Morelos	Unknown	Unknown	Unknown	Unknown
277	4.35	376	NA	Negative	0.17	ND	NA	22	M	Oaxaca	Unknown	Unknown	Unknown	Unknown
278	4.02	228	NA	Negative	0.68	ND	NA	35	M	Jalisco	Unknown	Unknown	Unknown	Unknown
279	4.43	763	NA	Negative	0.7	ND	NA	26	M	Morelos	Unknown	Unknown	Unknown	Unknown
280	5.03	441	NA	Negative	0.21	ND	NA	24	M	Morelos	Unknown	Unknown	Unknown	Unknown
281	4.95	86	NA	Negative	0.28	ND	NA	23	M	Morelos	Unknown	Unknown	Unknown	Unknown
282	5.35	386	NA	Negative	0.19	ND	NA	21	M	Jalisco	Unknown	Unknown	Unknown	Unknown
283	3.78	812	NA	Negative	0.37	ND	NA	43	M	DF	Unknown	Unknown	Unknown	Unknown
284	3.38	614	NA	Negative	0.39	ND	NA	26	M	Jalisco	Unknown	Unknown	Unknown	Unknown
285	5.07	49	NA	Negative	0.13	ND	NA	26	M	Jalisco	Unknown	Unknown	Unknown	Unknown
286	4.09	201	NA	Negative	0.29	ND	NA	48	M	Edo. Méx	Unknown	Unknown	Unknown	Unknown
287	3.99	267	NA	Negative	0.67	ND	NA	34	F	Edo. Méx	Unknown	Unknown	Unknown	Unknown
288	3.83	466	NA	Negative	1.72	ND	NA	58	M	Jalisco	Unknown	Unknown	Unknown	Unknown
289	4.34	380	NA	Negative	0.26	ND	NA	26	M	Jalisco	Unknown	Unknown	Unknown	Unknown
290	3.57	185	NA	Negative	0.75	ND	NA	37	M	DF	Unknown	Unknown	Unknown	Unknown
291	4.47	76	NA	Negative	0.18	ND	NA	29	M	DF	Unknown	Unknown	Unknown	Unknown
292	4.46	257	NA	Negative	0.37	ND	NA	35	M	DF	Unknown	Unknown	Unknown	Unknown
293	6.01	19	NA	Negative	0.09	ND	NA	25	F	Edo. Méx	Unknown	Unknown	Unknown	Unknown

294	5.30	192	NA	Negative	0.19	ND	NA	59	M	Morelos	Unknown	Unknown	Unknown	Unknown
295	5.24	61	NA	Negative	0.08	ND	NA	49	M	DF	Unknown	Unknown	Unknown	Unknown
296	4.38	761	NA	Negative	0.39	ND	NA	33	M	Sonora	Unknown	Unknown	Unknown	Unknown
297	4.61	565	NA	Negative	0.59	ND	NA	33	F	Morelos	Unknown	Unknown	Unknown	Unknown
298	5.68	341	NA	Negative	0.3	ND	NA	35	M	DF	Unknown	Unknown	Unknown	Unknown
299	4.31	585	NA	Negative	0.98	ND	NA	27	M	DF	Unknown	Unknown	Unknown	Unknown
300	4.26	42	NA	Negative	0.1	ND	NA	31	F	DF	Unknown	Unknown	Unknown	Unknown
301	5.04	256	NA	Negative	0.19	ND	NA	27	M	Edo. Méx	Unknown	Unknown	Unknown	Unknown
302	5.01	222	NA	Negative	0.09	ND	NA	35	M	Edo. Méx	Unknown	Unknown	Unknown	Unknown
303	4.91	740	NA	Negative	0.69	ND	NA	35	M	Puebla	Unknown	Unknown	Unknown	Unknown
304	4.48	319	NA	Negative	0.25	ND	NA	18	M	Edo. Méx	Unknown	Unknown	Unknown	Unknown
305	4.72	182	NA	Negative	0.27	ND	NA	26	M	Puebla	Unknown	Unknown	Unknown	Unknown
306	4.50	492	NA	Negative	0.48	ND	NA	21	M	Puebla	Unknown	Unknown	Unknown	Unknown
307	5.73	88	NA	Negative	0.11	ND	NA	Unknown	M	Puebla	Unknown	Unknown	Unknown	Unknown
308	4.50	999	NA	Negative	0.56	ND	NA	Unknown	F	DF	Unknown	Unknown	Unknown	Unknown
309	5.51	110	NA	Negative	0.13	ND	NA	33	M	DF	Unknown	Unknown	Unknown	Unknown
310	4.51	257	NA	Negative	0.4	ND	NA	38	M	Jalisco	Unknown	Unknown	Unknown	Unknown
311	3.54	826	NA	Negative	0.36	ND	NA	26	M	DF	Unknown	Unknown	Unknown	Unknown
312	4.75	266	NA	Negative	0.23	ND	NA	36	M	DF	Unknown	Unknown	Unknown	Unknown
313	5.03	129	NA	Negative	0.19	ND	NA	24	M	Puebla	Unknown	Unknown	Unknown	Unknown
314	6.10	140	NA	Negative	0.13	ND	NA	21	M	Edo. Méx	Unknown	Unknown	Unknown	Unknown
315	4.62	250	NA	Negative	0.28	ND	NA	26	M	Puebla	Unknown	Unknown	Unknown	Unknown
316	5.59	117	NA	Negative	0.2	ND	NA	49	M	Jalisco	Unknown	Unknown	Unknown	Unknown
317	5.50	41	NA	Negative	0.15	ND	NA	28	M	DF	Unknown	Unknown	Unknown	Unknown
318	4.89	292	NA	Negative	0.17	ND	NA	29	M	DF	Unknown	Unknown	Unknown	Unknown
319	4.78	283	NA	Negative	0.24	ND	NA	38	M	Edo. Méx	Unknown	Unknown	Unknown	Unknown
320	5.69	84	NA	Negative	0.14	ND	NA	28	M	Puebla	Unknown	Unknown	Unknown	Unknown
321	5.03	222	NA	Negative	0.37	ND	NA	38	M	DF	Unknown	Unknown	Unknown	Unknown
322	5.94	347	NA	Negative	0.25	ND	NA	36	M	DF	Unknown	Unknown	Unknown	Unknown
323	5.31	317	NA	Negative	0.31	ND	NA	31	M	Edo. Méx	Unknown	Unknown	Unknown	Unknown
324	4.53	139	NA	Negative	0.23	ND	NA	30	M	Puebla	Unknown	Unknown	Unknown	Unknown
325	5.98	28	NA	Negative	0.05	ND	NA	40	M	DF	Unknown	Unknown	Unknown	Unknown
326	5.53	171	NA	Negative	0.13	ND	NA	35	M	DF	Unknown	Unknown	Unknown	Unknown

327	4.87	89	NA	Negative	0.23	ND	NA	33	M	Puebla	Unknown	Unknown	Unknown	Unknown
328	5.40	474	NA	Negative	0.32	ND	NA	58	M	Edo. Méx	Unknown	Unknown	Unknown	Unknown
329	5.01	72	NA	Negative	0.16	ND	NA	24	M	Unknown	Unknown	Unknown	Unknown	Unknown
330	6.23	63	NA	Negative	0.15	ND	NA	19	M	Edo. Méx	Unknown	Unknown	Unknown	Unknown
331	4.14	350	NA	Negative	0.35	Late	NA	37	M	Michoacán	Single	Primary	Unemployed	MSM/IDU
332	5.58	484	NA	Negative	0.22	Late	NA	34	M	Michoacán	Unknown	Technician	Employed	MSM
333	3.69	420	NA	Negative	0.33	Late	NA	37	M	Morelos	Unknown	Unknown	Unknown	Unknown
334	4.91	357	NA	Negative	0.33	Recent	NA	34	F	Puebla	Married	High School	Unemployed	Heterosexual
335	3.97	225	NA	Negative	0.54	Recent	NA	24	F	Edo. Méx	Married	None	Unemployed	Heterosexual
336	3.79	1332	NA	Negative	1.45	Late	NA	Unknown	F	Colima	Unknown	Unknown	Unknown	Unknown
337	4.74	1017	NA	Negative	0.61	Late	NA	19	F	Colima	Free Union	High School	Unemployed	Unknown
338	4.84	211	NA	Negative	0.25	Late	NA	Unknown	M	Yucatán	Unknown	Unknown	Unknown	Unknown
339	4.84	124	NA	Negative	0.08	Late	NA	36	M	DF	Single	High School	Employed	MSM
340	5.70	300	NA	Negative	0.19	Recent	NA	31	M	Puebla	Single	High School	Employed	MSM
341	5.47	69	NA	Negative	0.16	Late	NA	35	F	puebla	Single	Primary	Employed	Heterosexual
342	5.51	72	NA	Negative	0.17	Late	NA	22	M	Puebla	Single	Technician	Unemployed	MSM
343	5.45	415	NA	Negative	0.16	Late	NA	32	M	Edo. Méx	Unknown	Unknown	Unknown	Unknown
344	5.47	211	NA	Negative	0.12	Late	NA	31	M	DF	Unknown	Unknown	Unknown	Unknown
345	3.13	466	NA	Negative	0.81	Late	NA	20	M	Guerrero	Single	Technician	Employed	MSM
346	5.61	157	NA	Negative	0.12	Late	NA	47	M	Edo. Méx	Single	Primary	Employed	Heterosexual
347	4.22	929	NA	Negative	1.08	Recent	NA	28	F	Puebla	Free Union	High School	Unemployed	Heterosexual
348	5.08	175	NA	Negative	0.17	Late	NA	52	M	DF	Married	Technician	Employed	MSM
349	5.17	684	NA	Negative	0.36	Recent	NA	19	F	Puebla	Single	High School	Unemployed	Heterosexual
350	5.90	62	NA	Negative	0.14	Late	NA	24	F	DF	Free Union	None	Unemployed	Heterosexual
351	5.23	36	NA	Negative	0.09	Late	NA	24	M	DF	Single	High School	Employed	MSM
352	5.98	114	NA	Negative	0.2	Late	NA	27	M	DF	Single	Technician	Student	MSM
353	6.07	188	NA	Negative	0.16	Late	NA	28	M	Oaxaca	Single	High School	Unemployed	MSM
354	5.21	267	NA	Negative	0.13	Recent	NA	21	F	Puebla	Free Union	High School	Unemployed	Heterosexual
355	5.37	51	NA	Negative	0.05	Late	NA	24	M	Puebla	Single	Technician	Employed	MSM
356	3.97	607	NA	Negative	0.37	Late	NA	21	M	Puebla	Free Union	High School	Employed	MSM
357	4.43	223	NA	Negative	0.18	Late	NA	33	M	Tijuana	Single	High School	Unemployed	MSM

358	4.12	595	NA	Negative	0.57	Recent	NA	27	M	Tijuana	Single	Technician	Employed	Bisexual
359	4.10	718	NA	Negative	0.5	Late	NA	40	M	Michoacán	Single	Technician	Employed	MSM
360	4.34	342	NA	Negative	0.5	Late	NA	23	M	Michoacán	Single	Technician	Employed	MSM
361	3.57	294	NA	Negative	0.79	Late	NA	44	F	Michoacán	Single	None	Employed	Heterosexual
362	5.45	279	NA	Negative	0.24	Late	NA	31	M	DF	Single	Technician	Unemployed	MSM
363	5.10	28	NA	Negative	0.01	Late	NA	34	M	DF	Single	Technician	Employed	MSM
364	5.82	74	NA	Negative	0.15	Late	NA	41	M	DF	Single	Posgraduate	Employed	Unknown
365	5.11	293	NA	Negative	0.52	Late	NA	30	F	Tijuana	Single	Primary	Unemployed	Heterosexual
366	5.05	271	NA	Negative	0.6	Recent	NA	42	M	Tijuana	Single	Primary	Employed	MSM
367	3.78	13	NA	Negative	0.05	Late	NA	30	M	Tijuana	Free Union	High School	Employed	Heterosexual
368	4.26	169	NA	Negative	0.18	Late	NA	32	F	Tijuana	Free Union	High School	Employed	Heterosexual
369	5.79	52	NA	Negative	0.07	Late	NA	45	F	Tijuana	Single	Primary	Unemployed	Heterosexual
370	3.89	283	NA	Negative	0.28	Late	NA	25	M	Tijuana	Free Union	Primary	Employed	Heterosexual
371	5.14	289	NA	Negative	0.16	Late	NA	30	M	Tijuana	Single	High School	Employed	MSM
372	5.23	265	NA	Negative	0.17	Late	NA	30	M	Puebla	Single	Technician	Employed	MSM
373	5.29	171	NA	Negative	0.08	Late	NA	32	M	Puebla	Single	High School	Employed	MSM
374	5.38	493	NA	Negative	0.35	Recent	NA	37	M	Tijuana	Single	High School	Unemployed	MSM
375	4.94	79	NA	Negative	0.07	Late	NA	43	M	Tijuana	Single	High School	Unemployed	Heterosexual
376	6.10	91	NA	Negative	0.07	Late	NA	55	M	Tijuana	Free Union	None	Unemployed	Heterosexual
377	4.62	385	NA	Negative	0.23	Recent	NA	23	M	Tijuana	Single	High School	Employed	MSM
378	5.70	179	NA	Negative	0.11	Late	NA	38	M	Tijuana	Single	High School	Unemployed	Heterosexual
379	6.29	8	NA	Negative	0.02	Late	NA	40	M	Tijuana	Single	Primary	Unemployed	Heterosexual
380	6.21	83	NA	Negative	0.06	Late	NA	40	F	Tijuana	Single	Primary	Unemployed	Heterosexual
381	3.26	453	NA	Negative	0.47	Recent	NA	19	M	Puebla	Single	High School	Student	MSM
382	4.96	356	NA	Negative	0.3	Recent	NA	26	F	Puebla	Free Union	Primary	Unemployed	Heterosexual
383	5.90	24	NA	Negative	0.12	Late	NA	25	M	Puebla	Free Union	Technician	Student	Heterosexual
384	5.99	215	NA	Negative	0.23	Late	NA	22	M	DF	Unknown	Technician	Student	MSM
385	4.69	271	NA	Negative	0.31	Late	NA	25	M	Tijuana	Single	High School	Employed	Heterosexual/IDU
386	5.29	71	NA	Negative	0.06	Late	NA	40	M	Tijuana	Free Union	High School	Unemployed	Heterosexual
387	4.76	69	NA	Negative	0.02	Late	NA	21	M	Tijuana	Single	None	Unemployed	MSM/IDU

388	3.85	277	NA	Negative	0.19	Late	NA	30	M	Tijuana	Single	High School	Employed	MSM
389	6.24	130	NA	Negative	0.32	Late	NA	34	M	Tijuana	Single	Primary	Unemployed	Heterosexual/IDU
390	4.18	648	NA	Negative	0.49	Recent	NA	20	M	Puebla	Single	High School	Unemployed	MSM
391	4.15	147	NA	Negative	0.1	Late	NA	29	M	Puebla	Married	High School	Employed	Heterosexual
392	5.92	145	NA	Negative	0.17	Late	NA	20	M	DF	Single	Technician	Unemployed	Heterosexual
393	4.97	890	NA	Negative	0.55	Recent	NA	16	F	Tijuana	Single	Primary	Unemployed	Heterosexual
394	4.77	313	NA	Negative	0.34	Late	NA	26	F	Quintana Roo	Free Union	Unknown	Unknown	Unknown
395	5.94	66	NA	Negative	0.04	Late	NA	23	M	Quintana Roo	Single	Unknown	Unknown	Unknown
396	4.86	38	NA	Negative	0.09	Late	NA	31	M	Tijuana	Free Union	High School	Unemployed	Heterosexual
397	4.56	265	NA	Negative	0.3	Late	NA	36	M	Tijuana	Free Union	Primary	Unemployed	Heterosexual
398	5.40	11	NA	Negative	0.05	Late	NA	44	F	Puebla	Married	Primary	Employed	Heterosexual
399	4.63	69	NA	Negative	0.04	Late	NA	Unknown	M	Unknown	Unknown	Unknown	Unknown	Unknown
400	5.19	77	NA	Negative	0.09	Late	NA	29	F	DF	Single	Primary	Unemployed	Heterosexual
401	5.44	225	NA	Negative	0.07	Late	NA	70	M	DF	Unknown	Unknown	Unknown	Unknown
402	5.73	65	NA	Negative	0.07	Late	NA	33	M	Tijuana	Single	High School	Unemployed	MSM
403	5.67	33	NA	Negative	0.02	Late	NA	49	M	DF	Unknown	Technician	Employed	MSM
404	5.62	43	NA	Negative	0.07	Late	NA	27	M	DF	Single	Technician	Employed	MSM
405	5.35	61	NA	Negative	0.22	Late	NA	18	M	DF	Single	High School	Unemployed	MSM
406	4.44	616	NA	Negative	0.41	Late	NA	24	M	DF	Single	Technician	Employed	MSM
407	5.47	50	NA	Negative	0.1	Late	NA	38	M	Tijuana	Single	Primary	Unemployed	Heterosexual
408	3.45	879	NA	Negative	0.45	Late	NA	23	M	Tijuana	Free Union	High School	Unemployed	Heterosexual
409	6.61	221	NA	Negative	0.74	Recent	NA	39	M	Tijuana	Single	High School	Unemployed	MSM
410	6.22	63	NA	Negative	0.3	Late	NA	21	F	Puebla	Free Union	Primary	Unemployed	Heterosexual
411	5.34	423	NA	Negative	0.3	Late	NA	26	F	Puebla	Single	High School	Unemployed	Heterosexual
412	4.17	275	NA	Negative	0.18	Recent	NA	23	M	Puebla	Single	High School	Employed	MSM
413	3.49	412	NA	Negative	0.24	Late	NA	24	M	Puebla	Single	High School	Student	MSM
414	6.83	628	NA	Negative	0.59	Recent	NA	36	M	Tijuana	Single	Technician	Employed	MSM
415	4.60	488	NA	Negative	0.29	Late	NA	36	M	Puebla	Free Union	Technician	Employed	Bisexual
416	6.37	10	NA	Negative	0.09	Late	NA	48	M	Tijuana	Single	Primary	Unemployed	Heterosexual
417	5.65	306	NA	Negative	0.24	Recent	NA	23	M	Puebla	Single	Technician	Student	MSM

418	4.47	155	NA	Negative	0.27	Late	NA	34	M	Puebla	Single	Technician	Employed	MSM
419	4.67	95	NA	Negative	0.07	Late	NA	22	F	Puebla	Free Union	Technician	Employed	Heterosexual
420	4.12	400	NA	Negative	0.25	Recent	NA	18	F	Edo. Méx	Free Union	High School	Unemployed	Heterosexual
421	4.50	373	NA	Negative	0.36	Recent	NA	29	M	Puebla	Single	Primary	Employed	MSM
422	5.40	264	NA	Negative	0.18	Recent	NA	26	M	Puebla	Single	Primary	Unemployed	MSM
423	4.17	415	NA	Negative	0.62	Late	NA	30	M	DF	Single	Technician	Employed	MSM
424	2.34	587	NA	Negative	0.39	Late	NA	17	M	Quintana Roo	Single	High School	Employed	MSM
425	3.65	206	NA	Negative	0.24	Late	NA	38	F	Puebla	Single	High School	Employed	MSM/IDU
426	4.52	498	NA	Negative	0.75	Late	NA	27	M	Puebla	Single	Technician	Employed	MSM
427	4.35	854	NA	Negative	0.45	Late	NA	39	F	Puebla	Married	High School	Employed	Heterosexual
428	4.45	182	NA	Negative	0.05	Late	NA	27	M	Puebla	Single	Technician	Student	MSM
429	5.62	208	NA	Negative	0.09	Late	NA	33	M	Puebla	Single	High School	Unknown	MSM
430	5.51	12	NA	Negative	0.05	Late	NA	23	M	Edo. Méx	Single	High School	Employed	MSM
431	4.85	254	NA	Negative	0.24	Late	NA	27	F	Puebla	Married	High School	Employed	Heterosexual
432	4.98	607	NA	Negative	1.18	Late	NA	23	M	Puebla	Single	Technician	Student	MSM
433	6.16	8	NA	Negative	0.03	Late	NA	37	M	DF	Single	High School	Employed	MSM
434	5.53	100	NA	Negative	0.23	Late	NA	34	F	Quintana Roo	Single	Primary	Unemployed	Unknown
435	6.36	23	NA	Negative	0.06	Late	NA	26	M	Quintana Roo	Single	High School	Unemployed	Unknown
436	4.35	767	NA	Negative	0.42	Late	NA	42	M	Quintana Roo	Single	High School	Employed	Unknown
437	5.18	516	NA	Negative	1.34	Late	NA	25	M	Quintana Roo	Single	High School	Employed	Unknown
438	5.16	245	NA	Negative	0.28	Recent	NA	23	M	Quintana Roo	Single	High School	Unemployed	Unknown
439	5.38	51	NA	Negative	0.04	Late	NA	40	M	Quintana Roo	Single	High School	Unemployed	Unknown
440	5.66	12	NA	Negative	0.03	Late	NA	31	M	Quintana Roo	Single	High School	Employed	Unknown
441	4.08	335	NA	Negative	0.21	Late	NA	38	M	Quintana Roo	Single	High School	Employed	Heterosexual
442	5.14	497	NA	Negative	0.29	Late	NA	20	M	Quintana Roo	Single	High School	Employed	MSM
443	5.03	209	NA	Negative	0.2	Late	NA	24	M	Quintana Roo	Single	Unknown	Unknown	Unknown
444	4.62	335	NA	Negative	0.41	Late	NA	21	M	Quintana Roo	Single	High School	Unemployed	Unknown

445	2.22	204	NA	Negative	0.43	Late	NA	55	M	Quintana Roo	Married	Primary	Employed	Heterosexual
446	4.81	457	NA	Negative	0.15	Late	NA	23	M	Quintana Roo	Single	High School	Employed	MSM
447	4.84	155	NA	Negative	0.27	Late	NA	35	M	Quintana Roo	Single	High School	Employed	Heterosexual
448	4.57	997	NA	Negative	0.57	Recent	NA	29	M	Quintana Roo	Single	High School	Employed	Unknown
449	4.73	283	NA	Negative	0.17	Late	NA	31	M	Quintana Roo	Unknown	High School	Employed	Unknown
450	5.68	209	NA	Negative	0.08	Recent	NA	29	M	Quintana Roo	Single	High School	Unemployed	MSM
451	6.73	329	NA	Negative	0.25	Recent	NA	26	M	Yucatán	Single	Technician	Employed	MSM
452	3.70	359	NA	Negative	0.21	Late	NA	43	M	Yucatán	Single	Technician	Unknown	Unknown
453	4.82	349	NA	Negative	0.11	Late	NA	40	M	Yucatán	Single	Posgraduate	Unknown	MSM
454	3.71	402	NA	Negative	0.39	Recent	NA	54	M	Yucatán	Single	High School	Employed	MSM
455	4.63	267	NA	Negative	0.26	Late	NA	47	M	Yucatán	Single	Technician	Employed	Unknown
456	6.18	46	NA	Negative	0.1	Late	NA	41	F	Tijuana	Single	High School	Unknown	Heterosexual
457	6.12	67	NA	Negative	0.29	Late	NA	25	M	Tijuana	Single	Unknown	Unknown	Unknown
458	4.28	5	NA	Negative	0.01	Late	NA	30	M	Tijuana	Single	High School	Unknown	Heterosexual
459	5.34	30	NA	Negative	0.05	Late	NA	34	M	Tijuana	Free Union	Primary	Unemployed	Heterosexual
460	5.95	73	NA	Negative	0.18	Late	NA	42	M	Tijuana	Single	High School	Unknown	Bisexual
461	5.14	4	NA	Negative	0.01	Late	NA	45	M	DF	Married	High School	Employed	Heterosexual
462	5.21	105	NA	Negative	0.08	Late	NA	39	M	DF	Single	Technician	Unemployed	MSM
463	3.91	427	NA	Negative	0.14	Late	NA	60	M	Tijuana	Single	High School	Unemployed	Heterosexual
464	5.44	205	NA	Negative	0.17	Late	NA	32	M	DF	Single	High School	Unemployed	MSM
465	5.02	330	NA	Negative	0.3	Late	NA	26	M	Puebla	Single	Technician	Unemployed	MSM
466	6.46	13	NA	Negative	0.04	Late	NA	34	M	Edo. Méx	Married	High School	Unemployed	Heterosexual
467	5.65	92	NA	Negative	0.04	Late	NA	32	M	Tijuana	Single	Primary	Unknown	Heterosexual
468	6.36	56	NA	Negative	0.12	Late	NA	23	F	Tijuana	Single	High School	Unemployed	Heterosexual
469	5.42	21	NA	Negative	0.07	Late	NA	40	M	Tijuana	Single	Unknown	Employed	Bisexual
470	5.81	41	NA	Negative	0.05	Late	NA	54	M	Tijuana	Single	High School	Unemployed	Heterosexual
471	4.90	832	NA	Negative	0.37	Recent	NA	21	M	Puebla	Single	High School	Unemployed	MSM
472	5.41	218	NA	Negative	0.14	Late	NA	76	F	Puebla	Single	Primary	Employed	Heterosexual
473	4.38	691	NA	Negative	0.49	Recent	NA	21	M	Puebla	Single	Technician	Employed	MSM
474	5.05	52	NA	Negative	0.06	Late	NA	66	M	Yucatán	Married	Technician	Unemployed	Bisexual

475	3.74	221	NA	Negative	0.17	Late	NA	37	M	Campeche	Single	High School	Employed	MSM
476	5.94	262	NA	Negative	0.07	Recent	NA	37	M	Campeche	Free Union	High School	Employed	Heterosexual
477	6.47	95	NA	Negative	0.21	Late	NA	27	M	Yucatán	Single	Posgraduate	Student	Bisexual
478	5.67	38	1.70	Low	0.08	Late	ND	38	M	Edo. Méx	Single	High School	Unemployed	Heterosexual
479	3.60	416	1.76	Low	0.47	Late	3	44	M	DF	Unknown	Posgraduate	Employed	MSM
480	5.08	61	2.13	Low	0.24	Late	3	33	M	DF	Unknown	High School	Unemployed	MSM
481	4.39	477	2.15	Low	0.42	Late	ND	23	M	Michoacán	Single	Technician	Student	MSM
482	4.91	367	2.18	Low	0.6	Recent	3	30	M	Edo. Méx	Single	Primary	Employed	MSM
483	6.21	169	2.19	Low	0.37	Late	2	20	M	DF	Single	High School	Student	MSM
484	4.54	12	2.24	Low	0.02	Late	ND	26	M	Tijuana	Single	High School	Unemployed	MSM
485	5.50	291	2.26	Low	0.29	Recent	ND	41	M	DF	Single	Technician	Employed	MSM
486	4.81	502	2.28	Low	0.73	Recent	ND	36	M	Edo. Méx	Unknown	Unknown	Unknown	Unknown
487	3.73	691	2.32	Low	0.32	Late	2	32	M	Michoacán	Married	High School	Unknown	Heterosexual
488	6.00	85	2.33	Low	0.12	ND	ND	32	M	Jalisco	Unknown	Unknown	Unknown	Unknown
489	2.75	336	2.33	Low	0.39	Late	ND	57	M	Quintana Roo	Single	Technician	Unemployed	Bisexual
490	3.80	749	2.36	Low	0.57	Recent	2	26	M	Puebla	Single	Technician	Employed	MSM
491	4.26	49	2.36	Low	0.09	Late	ND	33	F	Puebla	Free Union	Primary	Unemployed	Heterosexual
492	5.13	251	2.37	Low	0.22	Late	3	24	M	DF	Single	Technician	Unemployed	MSM
493	3.96	335	2.47	Low	0.48	Recent	3	21	M	Yucatán	Single	Technician	Student	Unknown
494	5.58	51	2.49	Low	0.21	Late	3	46	M	Puebla	Married	High School	Employed	Heterosexual
495	3.17	235	2.56	Low	0.37	Late	ND	25	M	Michoacán	Single	Technician	Employed	MSM
496	4.09	310	2.57	Low	0.46	Late	3	38	F	Michoacán	Single	Primary	Unknown	Heterosexual
497	5.23	140	2.60	Low	0.06	Late	2b/3	31	M	Tijuana	Single	Technician	Unknown	MSM
498	3.41	686	2.63	Low	0.73	Recent	2	22	M	Michoacán	Single	Technician	Student	MSM
499	5.38	27	2.70	Low	0.05	Late	3	30	M	DF	Single	High School	Unemployed	MSM
500	5.23	19	2.70	Low	0.06	ND	2	31	M	Morelos	Unknown	Unknown	Unknown	Unknown
501	5.79	37	2.71	Low	0.02	Late	ND	42	M	Quintana Roo	Single	Unknown	Unknown	Unknown
502	5.63	117	2.75	Low	0.12	ND	2	36	M	Edo. Méx	Unknown	Unknown	Unknown	Unknown
503	5.17	34	2.75	Low	0.06	ND	2a/3	35	M	Veracruz	Unknown	Unknown	Unknown	Unknown
504	5.35	53	2.78	Low	0.09	Late	3	48	M	Tijuana	Married	Primary	Unknown	Heterosexual
505	3.59	1031	2.78	Low	0.47	Recent	3	21	F	Quintana Roo	Free Union	High School	Unemployed	Heterosexual

506	5.31	150	2.78	Low	0.42	Late	2	30	M	Edo. Méx	Free Union	High School	Unemployed	Bisexual
507	5.21	237	2.80	Low	0.1	ND	2a/3	33	M	DF	Unknown	Unknown	Unknown	Unknown
508	3.97	977	2.80	Low	0.44	Recent	3	25	F	Puebla	Single	High School	Unemployed	Heterosexual
509	5.04	611	2.83	Low	0.5	ND	ND	27	M	Morelos	Unknown	Unknown	Unknown	Unknown
510	5.38	170	2.84	Low	0.08	Late	3	49	M	Tijuana	Free Union	High School	Unknown	Heterosexual
511	6.14	146	2.85	Low	0.21	Late	ND	44	M	DF	Single	Technician	Unemployed	MSM
512	4.61	326	2.86	Low	0.45	ND	3	38	M	DF	Unknown	Unknown	Unknown	Unknown
513	5.34	14	2.88	Low	0.05	ND	2a/3	32	M	Edo. Méx	Unknown	Unknown	Unknown	Unknown
514	4.86	188	2.89	Low	0.12	Late	3	37	M	DF	Unknown	Unknown	Unknown	Unknown
515	4.91	411	2.89	Low	0.36	Late	2	39	M	DF	Free Union	High School	Employed	Heterosexual
516	5.64	719	2.90	Low	0.23	Recent	2b/3	27	M	Tijuana	Free Union	Technician	Student	Heterosexual
517	5.05	259	2.92	Low	0.18	ND	2a/3	31	M	DF	Unknown	Unknown	Unknown	Unknown
518	5.37	60	2.93	Low	0.03	Late	ND	29	M	Quintana Roo	Free Union	High School	Employed	Bisexual
519	4.45	309	2.93	Low	0.37	Late	3	58	M	Puebla	Single	Technician	Unemployed	MSM
520	5.84	18	2.94	Low	0.05	ND	ND	46	M	Veracruz	Unknown	Unknown	Unknown	Unknown
521	5.53	83	2.95	Low	0.11	ND	2a/3	33	M	Edo. Méx	Unknown	Unknown	Unknown	Unknown
522	4.38	740	2.99	Low	0.82	Recent	3	28	M	Edo. Méx	Unknown	Unknown	Unknown	Unknown
523	4.89	178	2.99	Low	0.24	ND	ND	39	F	Edo. Méx	Unknown	Unknown	Unknown	Unknown
524	5.08	263	2.99	Low	0.21	ND	ND	31	M	Puebla	Unknown	Unknown	Unknown	Unknown
525	5.54	98	2.99	Low	0.16	Late	3	34	M	Tijuana	Single	High School	Unknown	Heterosexual
526	5.08	74	3.00	Low	0.12	ND	3	27	M	DF	Unknown	Unknown	Unknown	Unknown
527	3.12	467	3.03	Low	0.36	Recent	ND	21	M	Puebla	Single	Technician	Student	MSM
528	5.33	390	3.03	Low	0.31	Recent	ND	45	M	Puebla	Married	High School	Employed	Heterosexual
529	4.01	456	3.05	Low	0.61	ND	2b/3	19	M	Jalisco	Unknown	Unknown	Unknown	Unknown
530	4.26	670	3.06	Low	0.42	ND	1	33	M	Edo. Méx	Unknown	Unknown	Unknown	Unknown
531	5.72	217	3.06	Low	0.34	ND	3	36	F	Morelos	Unknown	Unknown	Unknown	Unknown
532	5.26	157	3.06	Low	0.19	Late	3	29	M	DF	Single	Technician	Employed	MSM
533	4.66	234	3.07	Low	0.12	Late	3	37	F	DF	Single	Technician	Unemployed	Heterosexual
534	5.05	151	3.12	Low	0.11	ND	2	51	F	Morelos	Unknown	Unknown	Unknown	Unknown
535	5.80	69	3.14	Low	0.1	ND	ND	44	F	Morelos	Unknown	Unknown	Unknown	Unknown
536	4.90	43	3.16	Low	0.05	ND	2	28	M	Puebla	Unknown	Unknown	Unknown	Unknown

537	5.02	147	3.17	Low	0.09	Late	ND	38	M	DF	Single	Posgraduate	Employed	MSM
538	4.31	624	3.20	Low	0.41	Late	3	33	M	Michoacán	Single	Technician	Unemployed	MSM
539	4.71	477	3.20	Low	0.81	Recent	3	20	M	Puebla	Single	Technician	Student	MSM
540	3.42	706	3.21	Low	1.35	ND	ND	33	M	Nuevo León	Unknown	Unknown	Unknown	Unknown
541	5.20	527	3.21	Low	0.49	Late	2b/3	30	M	Quintana Roo	Free Union	High School	Employed	Unknown
542	2.52	59	3.22	Low	0.08	Late	3	29	M	Quintana Roo	Free Union	High School	Employed	Heterosexual
543	5.51	456	3.23	Low	0.11	ND	2	21	M	Edo. Méx	Unknown	Unknown	Unknown	Unknown
544	4.50	391	3.23	Low	0.43	Recent	ND	22	F	Puebla	Free Union	Technician	Unemployed	Heterosexual
545	5.08	203	3.24	Low	0.13	Late	ND	30	M	Yucatán	Single	Technician	Employed	MSM
546	5.10	62	3.25	Low	0.11	ND	2a/3	35	M	Edo. Méx	Unknown	Unknown	Unknown	Unknown
547	5.20	232	3.27	Low	0.31	ND	ND	28	M	Morelos	Unknown	Unknown	Unknown	Unknown
548	5.32	289	3.28	Low	0.25	ND	ND	48	F	Jalisco	Unknown	Unknown	Unknown	Unknown
549	5.20	188	3.28	Low	0.19	ND	3	28	F	Jalisco	Unknown	Unknown	Unknown	Unknown
550	5.64	290	3.28	Low	0.13	ND	2a/3	76	M	Morelos	Unknown	Unknown	Unknown	Unknown
551	5.64	28	3.28	Low	0.07	Late	3	26	M	Puebla	Single	High School	Employed	MSM
552	4.28	385	3.29	Low	0.81	ND	ND	25	M	Morelos	Unknown	Unknown	Unknown	Unknown
553	6.00	10	3.29	Low	0.05	ND	ND	29	M	Edo. Méx	Unknown	Unknown	Unknown	Unknown
554	5.15	202	3.29	Low	0.17	ND	2a/3	23	F	Edo. Méx	Unknown	Unknown	Unknown	Unknown
555	4.84	285	3.30	Low	0.44	ND	ND	23	M	Morelos	Unknown	Unknown	Unknown	Unknown
556	4.90	211	3.33	Low	0.15	ND	2a/3	29	M	Puebla	Unknown	Unknown	Unknown	Unknown
557	5.45	63	3.34	Low	0.11	ND	ND	28	M	Nuevo León	Unknown	Unknown	Unknown	Unknown
558	4.51	332	3.35	Low	0.21	Late	3	24	F	Quintana Roo	Free Union	High School	Unemployed	Heterosexual
559	3.99	122	3.35	Low	0.27	ND	3	31	M	Edo. Méx	Unknown	Unknown	Unknown	Unknown
560	4.49	3	3.36	Low	0.02	Late	2a/3	27	M	Quintana Roo	Single	High School	Unemployed	MSM
561	5.64	25	3.36	Low	0.06	ND	ND	29	M	Jalisco	Unknown	Unknown	Unknown	Unknown
562	6.34	32	3.38	Low	0.06	Late	ND	70	M	DF	Single	Technician	Unemployed	Heterosexual
563	4.87	364	3.39	Low	0.24	ND	ND	27	M	DF	Unknown	Unknown	Unknown	Unknown
564	4.80	193	3.39	Low	0.16	Late	3	24	M	Puebla	Single	Technician	Employed	MSM
565	3.80	412	3.39	Low	0.32	Late	3	20	M	DF	Single	Technician	Employed	MSM
566	5.32	8	3.40	Low	0.1	ND	ND	44	M	Jalisco	Unknown	Unknown	Unknown	Unknown

567	5.41	174	3.40	Low	0.09	ND	2	39	M	Morelos	Unknown	Unknown	Unknown	Unknown
568	5.27	115	3.40	Low	0.21	Late	2a/3	33	M	Puebla	Single	Primary	Employed	Heterosexual
569	5.00	59	3.40	Low	0.12	ND	2b/3	54	M	Edo. Méx	Unknown	Unknown	Unknown	Unknown
570	3.43	517	3.41	Low	0.33	ND	ND	54	M	Puebla	Unknown	Unknown	Unknown	Unknown
571	4.32	447	3.41	Low	0.77	ND	ND	23	M	Veracruz	Unknown	Unknown	Unknown	Unknown
572	4.53	348	3.42	Low	0.33	Recent	3	20	M	Tijuana	Single	High School	Employed	MSM
573	5.92	127	3.43	Low	0.19	Late	3	28	M	Puebla	Free Union	High School	Unemployed	Heterosexual
574	5.72	223	3.43	Low	0.23	ND	2	24	M	DF	Unknown	Unknown	Unknown	Unknown
575	5.15	140	3.44	Low	0.21	Late	ND	28	M	Edo. Méx	Single	High School	Unemployed	Unknown
576	5.03	155	3.44	Low	0.18	ND	3	30	M	Edo. Méx	Unknown	Unknown	Unknown	Unknown
577	4.28	334	3.45	Low	0.37	ND	ND	22	M	Morelos	Unknown	Unknown	Unknown	Unknown
578	5.15	409	3.45	Low	0.22	ND	2	18	M	Puebla	Unknown	Unknown	Unknown	Unknown
579	5.87	25	3.45	Low	0.06	Late	3	17	F	Tijuana	Single	Primary	Unemployed	Unknown
580	3.48	140	3.47	Low	0.4	ND	ND	32	F	Jalisco	Unknown	Unknown	Unknown	Unknown
581	5.42	15	3.47	Low	0.06	ND	ND	35	M	Edo. Méx	Unknown	Unknown	Unknown	Unknown
582	5.25	59	3.47	Low	0.07	Late	3	43	M	Puebla	Single	Technician	Employed	MSM
583	4.60	114	3.48	Low	0.15	ND	2b/3	40	M	Morelos	Unknown	Unknown	Unknown	Unknown
584	6.07	275	3.49	Low	0.1	Recent	3	34	M	Puebla	Free Union	Primary	Unemployed	Heterosexual
585	2.62	241	3.51	Low	0.83	Late	2b/3	27	M	Puebla	Single	High School	Unemployed	Heterosexual
586	3.24	9	3.51	Low	0.03	Late	3	27	M	Quintana Roo	Free Union	Primary	Employed	Heterosexual
587	3.71	496	3.51	Low	0.54	ND	ND	21	M	Veracruz	Unknown	Unknown	Unknown	Unknown
588	5.17	416	3.52	Low	0.31	Recent	3	25	M	Puebla	Single	High School	Employed	Bisexual
589	6.05	77	3.54	Low	0.15	Late	3	26	M	Edo. Méx	Single	Technician	Employed	Unknown
590	4.13	531	3.54	Low	0.43	Recent	3	40	M	Puebla	Single	High School	Employed	MSM
591	5.34	119	3.55	Low	0.09	Late	2b/3	27	M	Puebla	Single	Technician	Unemployed	MSM
592	4.75	67	3.55	Low	0.12	Late	ND	38	M	DF	Single	Technician	Unemployed	MSM/IDU
593	4.21	456	3.56	Low	0.25	ND	3	23	M	Veracruz	Unknown	Unknown	Unknown	Unknown
594	4.88	910	3.57	Low	0.77	Recent	ND	34	M	Puebla	Single	Technician	Employed	MSM
595	5.78	400	3.58	Low	0.35	Late	3	32	M	Quintana Roo	Single	Technician	Employed	MSM
596	4.80	223	3.59	Low	0.14	Late	2b/3	28	M	Puebla	Single	High School	Employed	MSM
597	5.44	265	3.59	Low	0.16	Recent	2a/3	18	M	Puebla	Single	High School	Unemployed	MSM
598	5.53	75	3.60	Low	0.1	Late	3	55	M	Edo. Méx	Unknown	Unknown	Unknown	Unknown

599	4.69	363	3.64	Low	0.58	Recent	ND	28	M	Puebla	Single	Technician	Employed	MSM
600	5.72	379	3.65	Low	0.22	Recent	ND	42	M	DF	Single	Posgraduate	Employed	MSM
601	5.37	146	3.68	Low	0.14	ND	3	27	M	Jalisco	Unknown	Unknown	Unknown	Unknown
602	5.85	208	3.70	Low	0.11	Late	3	83	M	DF	Unknown	Unknown	Unknown	Unknown
603	5.06	7	3.71	Low	0.01	Late	3	30	F	Tijuana	Unknown	Unknown	Unknown	Unknown
604	3.82	359	3.71	Low	0.36	ND	2a/3	21	F	Veracruz	Unknown	Unknown	Unknown	Unknown
605	5.39	78	3.72	Low	0.04	Late	ND	27	F	Tijuana	Single	Primary	Unemployed	Heterosexual
606	5.46	16	3.72	Low	0.02	ND	3	26	F	Edo. Méx	Unknown	Unknown	Unknown	Unknown
607	3.76	518	3.73	Low	0.58	Recent	2b/3	23	M	DF	Single	Technician	Employed	Unknown
608	4.87	418	3.73	Low	0.8	ND	2	44	M	Edo. Méx	Unknown	Unknown	Unknown	Unknown
609	4.80	510	3.74	Low	0.81	ND	2	49	M	DF	Unknown	Unknown	Unknown	Unknown
610	5.08	145	3.75	Low	0.26	ND	ND	34	M	Edo. Méx	Unknown	Unknown	Unknown	Unknown
611	4.64	44	3.75	Low	0.03	Late	ND	34	M	Tijuana	Unknown	High School	Employed	Heterosexual
612	4.71	151	3.75	Low	0.1	ND	2a/3	25	M	Morelos	Unknown	Unknown	Unknown	Unknown
613	4.41	741	3.76	Low	0.93	Recent	3	22	M	DF	Single	High School	Unemployed	MSM
614	4.68	65	3.80	Low	0.1	ND	2	24	M	veracruz	Unknown	Unknown	Unknown	Unknown
615	3.87	600	3.80	Low	1.16	Recent	ND	36	M	Tijuana	Single	Technician	Unknown	MSM
616	6.12	119	3.81	Low	0.14	ND	3	71	M	Morelos	Unknown	Unknown	Unknown	Unknown
617	1.72	544	3.82	Low	0.72	Late	ND	4	M	Quintana Roo	Single	Kinder	Student	Unknown
618	4.52	108	3.82	Low	0.06	Late	ND	26	M	Puebla	Single	Technician	Unemployed	MSM
619	4.12	642	3.84	Low	0.48	Late	ND	23	M	Quintana Roo	Single	Unknown	Unknown	Unknown
620	4.38	274	3.85	Low	0.19	ND	3	41	M	Veracruz	Unknown	Unknown	Unknown	Unknown
621	5.77	66	3.86	Low	0.05	Late	ND	40	M	DF	Married	Technician	Employed	Unknown
622	4.95	13	3.86	Low	0.03	ND	ND	32	M	Edo. Méx	Unknown	Unknown	Unknown	Unknown
623	4.11	299	3.86	Low	0.16	ND	3	36	M	Jalisco	Unknown	Unknown	Unknown	Unknown
624	5.60	198	3.86	Low	0.19	Late	3	29	M	Quintana Roo	Single	High School	Employed	Unknown
625	3.74	560	3.87	Low	0.29	Recent	3	21	M	Quintana Roo	Free Union	Primary	Unemployed	Heterosexual
626	4.50	108	3.88	Low	0.08	ND	3	42	M	DF	Unknown	Unknown	Unknown	Unknown
627	5.10	228	3.88	Low	0.28	ND	2	34	F	Morelos	Unknown	Unknown	Unknown	Unknown
628	1.69	428	3.92	Low	0.61	Late	ND	26	F	Michoacán	Free Union	High School	Employed	Heterosexual
629	6.40	55	3.94	Low	0.03	Late	2	43	M	Tijuana	Married	Primary	Unemployed	Heterosexual

630	4.55	266	3.94	Low	0.23	ND	ND	29	M	Jalisco	Unknown	Unknown	Unknown	Unknown
631	4.72	23	3.95	Low	0.18	ND	3	32	F	Edo. Méx	Unknown	Unknown	Unknown	Unknown
632	3.83	587	3.99	Low	0.4	Late	3	26	M	Tijuana	Single	Posgraduate	Employed	MSM
633	4.25	683	4.03	Low	1.26	Late	ND	Unknown	F	Unknown	Unknown	Unknown	Unknown	Unknown
634	5.69	703	4.04	Low	0.23	Recent	2	24	M	Edo. Méx	Unknown	Unknown	Unknown	Unknown
635	4.56	210	4.06	Low	0.2	ND	3	23	M	Veracruz	Unknown	Unknown	Unknown	Unknown
636	4.54	330	4.07	Low	0.71	ND	2	22	M	Veracruz	Unknown	Unknown	Unknown	Unknown
637	4.83	165	4.08	Low	0.57	Late	3	32	M	DF	Single	Technician	Employed	Accidente ocupacional
638	4.64	176	4.09	Low	0.17	ND	ND	30	M	Edo. Méx	Unknown	Unknown	Unknown	Unknown
639	4.69	203	4.14	Low	0.34	Recent	ND	17	M	Puebla	Single	Primary	Employed	Bisexual
640	5.30	46	4.18	Low	0.06	ND	2a/3	35	M	Jalisco	Unknown	Unknown	Unknown	Unknown
641	4.00	80	4.26	Low	0.11	Late	3	41	M	Tijuana	Single	Primary	Unemployed	Heterosexual
642	4.80	805	4.30	Low	0.72	Late	3	41	M	Michoacán	Free Union	High School	Unknown	MSM
643	5.55	5	4.33	Low	0.28	Late	3	27	M	Quintana Roo	Single	High School	Employed	MSM
644	4.46	470	4.39	Low	0.44	Recent	3	26	M	Puebla	Single	High School	Employed	MSM
645	5.85	79	4.42	Low	0.17	ND	ND	37	M	Morelos	Unknown	Unknown	Unknown	Unknown
646	4.40	429	4.43	Low	0.33	Recent	3	19	M	Quintana Roo	Single	Technician	Unemployed	MSM
647	3.98	1391	4.46	Low	0.67	Late	3	51	M	Quintana Roo	Single	None	Unemployed	Heterosexual
648	4.68	753	4.48	Low	0.8	Late	3	30	M	Quintana Roo	Single	Technician	Employed	MSM
649	5.25	25	4.52	Low	0.04	Late	3	38	M	Quintana Roo	Single	High School	Unemployed	Unknown
650	5.35	153	4.53	Low	0.33	ND	3	33	M	Jalisco	Unknown	Unknown	Unknown	Unknown
651	3.03	360	4.55	Low	0.35	Late	3	40	F	Michoacán	Married	Primary	Unemployed	Heterosexual
652	4.52	146	4.55	Low	0.14	Late	2a/3	42	M	Tlaxcala	Unknown	Unknown	Unknown	Unknown
653	4.18	158	4.61	Low	0.26	ND	2	33	M	Jalisco	Unknown	Unknown	Unknown	Unknown
654	5.41	62	4.63	Low	0.09	ND	3	27	F	Michoacán	Unknown	Unknown	Unknown	Unknown
655	4.55	593	4.69	Low	0.73	Recent	3	25	M	DF	Single	High School	Employed	MSM
656	4.65	109	4.72	Low	0.08	Late	3	27	M	DF	Free Union	High School	Employed	Heterosexual/IDU
657	5.08	323	4.72	Low	0.15	Recent	3	29	M	Puebla	Single	Technician	Employed	MSM
658	5.31	322	4.72	Low	0.14	Late	3	23	M	DF	Unknown	Unknown	Unknown	Unknown

659	5.16	162	4.75	Low	0.21	Late	ND	39	M	Jalisco	Single	Technician	Employed	MSM
660	5.75	50	4.75	Low	0.04	Late	ND	25	M	DF	Single	Technician	Student	MSM
661	3.85	182	4.76	Low	0.19	Late	ND	46	M	DF	Single	High School	Unemployed	MSM
662	4.58	587	4.77	Low	0.57	Recent	3	19	M	DF	Single	High School	Unemployed	MSM
663	4.24	520	4.78	Low	0.5	Recent	2	55	M	Jalisco	Free Union	High School	Employed	Heterosexual
664	5.23	152	4.78	Low	0.12	ND	ND	29	F	Morelos	Unknown	Unknown	Unknown	Unknown
665	4.14	721	4.78	Low	1.15	Late	2a/3	44	F	Tabasco	Unknown	Unknown	Unknown	Unknown
666	3.90	296	4.79	Low	0.42	Recent	ND	28	M	Puebla	Single	Technician	Unemployed	MSM
667	2.13	1523	4.81	Low	1.03	Late	2	43	F	Jalisco	Married	High School	Employed	Heterosexual
668	5.79	44	4.82	Low	0.52	Late	3	40	M	Jalisco	Married	Primary	Employed	Bisexual
669	4.55	115	4.90	Low	0.16	Late	ND	29	F	Edo. Méx	Free Union	High School	Employed	Unknown
670	6.00	14	5.00	Low	0.02	ND	ND	40	M	Veracruz	Unknown	Unknown	Unknown	Unknown
671	5.92	342	5.02	Low	0.35	ND	1	48	M	Veracruz	Unknown	Unknown	Unknown	Unknown
672	4.78	319	5.05	Low	0.18	Recent	3	33	M	Tijuana	Single	High School	Employed	MSM
673	3.48	544	5.05	Low	0.48	Recent	ND	19	M	Puebla	Single	High School	Employed	MSM
674	5.03	45	5.16	High	0.07	ND	ND	27	M	Veracruz	Unknown	Unknown	Unknown	Unknown
675	4.74	412	5.17	High	0.59	ND	2	33	M	Edo. Méx	Unknown	Unknown	Unknown	Unknown
676	4.80	261	5.19	High	ND	ND	3	42	M	Jalisco	Unknown	Unknown	Unknown	Unknown
677	5.26	131	5.22	High	0.11	ND	2	33	M	DF	Unknown	Unknown	Unknown	Unknown
678	4.96	119	5.23	High	0.15	Late	3	34	M	DF	Single	High School	Employed	MSM
679	5.69	148	5.23	High	0.1	Late	3	29	F	DF	Free Union	High School	Employed	Heterosexual
680	4.84	485	5.30	High	0.36	ND	ND	19	M	Puebla	Unknown	Unknown	Unknown	Unknown
681	4.87	341	5.37	High	0.27	ND	3	19	M	Veracruz	Unknown	Unknown	Unknown	Unknown
682	4.10	385	5.40	High	0.51	ND	3	33	M	DF	Unknown	Unknown	Unknown	Unknown
683	5.06	119	5.41	High	0.11	ND	2	27	M	Edo. Méx	Unknown	Unknown	Unknown	Unknown
684	4.70	206	5.46	High	0.33	ND	3	22	M	DF	Unknown	Unknown	Unknown	Unknown
685	4.79	351	5.53	High	0.18	ND	3	28	M	Morelos	Unknown	Unknown	Unknown	Unknown
686	3.23	632	5.60	High	0.76	Recent	3	38	F	Tijuana	Married	High School	Employed	Heterosexual
687	4.47	23	5.61	High	0.04	ND	3	39	M	Morelos	Unknown	Unknown	Unknown	Unknown
688	5.06	268	5.64	High	0.21	Recent	3	28	M	Puebla	Single	High School	Employed	MSM
689	4.89	376	5.69	High	0.17	Late	3	40	M	Quintana Roo	Free Union	Primary	Unemployed	Unknown
690	4.79	288	5.71	High	0.64	Late	3	28	M	Puebla	Single	Technician	Employed	MSM

691	4.88	233	5.71	High	0.11	ND	2	27	M	DF	Unknown	Unknown	Unknown	Unknown
692	4.61	1407	5.72	High	1.11	Recent	3	26	F	Tijuana	Single	Primary	Unemployed	Heterosexual
693	4.89	111	5.76	High	0.14	ND	2a/3	30	M	Edo. Méx	Unknown	Unknown	Unknown	Unknown
694	4.67	319	5.76	High	0.17	Recent	2	21	F	Puebla	Free Union	High School	Employed	Heterosexual
695	2.39	235	5.81	High	0.18	Late	ND	37	F	Quintana Roo	Single	Unknown	Unknown	Unknown
696	4.57	511	5.81	High	0.14	Recent	3	29	M	Quintana Roo	Single	High School	Employed	MSM
697	5.49	408	5.81	High	0.26	ND	3	22	M	Edo. Méx	Unknown	Unknown	Unknown	Unknown
698	4.70	358	5.83	High	0.29	Recent	3	19	M	Yucatán	Single	High School	Student	MSM
699	5.09	438	5.84	High	0.74	Recent	2	34	M	DF	Single	Technician	Employed	MSM
700	3.84	771	5.89	High	0.29	Recent	3	25	M	DF	Single	Posgraduate	Student	MSM
701	4.85	573	5.91	High	0.17	ND	2	35	M	DF	Unknown	Unknown	Unknown	Unknown
702	3.21	456	5.94	High	0.22	Late	3	37	M	Puebla	Married	High School	Employed	Heterosexual
703	3.29	403	5.94	High	0.24	Recent	3	27	M	Puebla	Single	Technician	Employed	MSM
704	5.13	255	5.97	High	0.25	ND	2	25	M	Morelos	Unknown	Unknown	Unknown	Unknown
705	2.13	400	5.98	High	0.78	Late	3	42	M	Tijuana	Single	None	Unemployed	MSM
706	5.96	790	6.01	High	0.7	ND	2a/3	29	F	Puebla	Unknown	Unknown	Unknown	Unknown
707	4.88	261	6.04	High	0.39	ND	3	24	M	DF	Unknown	Unknown	Unknown	Unknown
708	4.14	406	6.08	High	0.49	Recent	ND	23	M	Quintana Roo	Single	Technician	Employed	Unknown
709	3.87	266	6.13	High	0.29	ND	2	32	M	Morelos	Unknown	Unknown	Unknown	Unknown
710	4.14	118	6.16	High	0.03	Late	ND	45	M	Tijuana	Single	High School	Employed	Heterosexual
711	4.96	261	6.17	High	0.11	Late	3	24	M	Quintana Roo	Single	Technician	Student	Unknown
712	4.04	644	6.23	High	0.53	Recent	3	21	M	Tijuana	Free Union	High School	Student	Bisexual
713	4.81	287	6.26	High	0.25	Late	ND	30	M	DF	Unknown	Unknown	Unknown	Unknown
714	4.93	441	6.27	High	0.52	Recent	ND	22	M	Quintana Roo	Single	High School	Unemployed	MSM
715	5.08	382	6.28	High	0.22	ND	ND	25	M	DF	Unknown	Unknown	Unknown	Unknown
716	4.83	295	6.35	High	0.33	Late	2	41	M	DF	Single	Posgraduate	Employed	Bisexual
717	4.65	377	6.38	High	0.72	ND	2	24	M	DF	Unknown	Unknown	Unknown	Unknown
718	4.47	509	6.41	High	0.56	Late	ND	31	F	Quintana Roo	Unknown	Primary	Unemployed	Unknown
719	4.20	97	6.47	High	0.15	ND	3	28	F	Chiapas	Unknown	Unknown	Unknown	Unknown
720	4.43	302	6.47	High	0.2	Late	3	Unknown	M	Puebla	Unknown	Unknown	Unknown	Unknown

721	4.09	343	6.49	High	0.18	Late	2	30	M	Tijuana	Married	Technician	Employed	Heterosexual
722	5.28	259	6.50	High	0.17	ND	ND	27	M	Veracruz	Unknown	Unknown	Unknown	Unknown
723	4.45	193	6.50	High	0.14	ND	3	39	F	Morelos	Unknown	Unknown	Unknown	Unknown
724	4.81	313	6.51	High	0.26	ND	3	42	M	Veracruz	Unknown	Unknown	Unknown	Unknown
725	5.04	341	6.63	High	0.64	ND	3	28	M	Morelos	Unknown	Unknown	Unknown	Unknown
726	5.01	267	6.63	High	0.43	ND	ND	21	M	Morelos	Unknown	Unknown	Unknown	Unknown
727	4.74	330	6.64	High	0.29	Late	ND	22	M	Yucatán	Single	Technician	Employed	MSM
728	5.26	241	6.67	High	0.17	Late	2	28	M	DF	Single	Technician	Employed	MSM
729	4.04	360	6.68	High	0.39	ND	3	27	M	Veracruz	Unknown	Unknown	Unknown	Unknown
730	3.91	243	6.72	High	0.6	ND	2	24	M	Veracruz	Unknown	Unknown	Unknown	Unknown
731	4.19	310	6.73	High	0.38	ND	3	26	F	Edo. Méx	Unknown	Unknown	Unknown	Unknown
732	5.56	340	6.80	High	0.13	ND	3	29	M	DF	Unknown	Unknown	Unknown	Unknown
733	5.05	298	6.80	High	0.45	Late	3	21	M	Puebla	Single	Technician	Student	MSM
734	4.96	219	6.82	High	0.23	ND	3	31	M	DF	Unknown	Unknown	Unknown	Unknown
735	3.40	857	6.82	High	0.96	ND	3	26	F	Edo. Méx	Unknown	Unknown	Unknown	Unknown
736	4.68	506	6.83	High	0.33	ND	ND	28	M	Edo. Méx	Unknown	Unknown	Unknown	Unknown
737	4.47	254	6.87	High	0.62	ND	2	31	M	Edo. Méx	Unknown	Unknown	Unknown	Unknown
738	4.59	411	6.87	High	0.74	Recent	3	19	M	Puebla	Single	Technician	Student	MSM
739	4.35	722	6.87	High	0.48	ND	3	36	M	Veracruz	Unknown	Unknown	Unknown	Unknown
740	4.36	185	6.89	High	0.08	ND	3	37	M	Edo. Méx	Unknown	Unknown	Unknown	Unknown
741	4.50	341	6.91	High	0.46	ND	2	28	M	Veracruz	Unknown	Unknown	Unknown	Unknown
742	3.00	729	6.91	High	1.26	Late	3	31	F	Edo. Méx	Unknown	Unknown	Unknown	Unknown
743	5.77	450	6.91	High	0.55	Recent	2	24	M	DF	Single	High School	Employed	MSM
744	4.03	183	6.93	High	0.45	ND	2a/3	40	F	Jalisco	Unknown	Unknown	Unknown	Unknown
745	4.03	300	6.99	High	0.33	ND	3	23	M	Edo. Méx	Unknown	Unknown	Unknown	Unknown
746	5.01	115	6.99	High	0.06	ND	ND	34	M	Edo. Méx	Unknown	Unknown	Unknown	Unknown
747	4.16	678	7.02	High	0.71	Late	ND	33	M	Puebla	Free Union	Primary	Employed	Heterosexual
748	3.66	701	7.08	High	0.44	Late	3	44	M	Jalisco	Single	Technician	Unemployed	Bisexual
749	5.28	344	7.08	High	0.46	ND	3	28	M	DF	Unknown	Unknown	Unknown	Unknown
750	4.57	358	7.13	High	0.55	ND	3	22	M	Edo. Méx	Unknown	Unknown	Unknown	Unknown
751	4.15	474	7.14	High	0.46	ND	2	Unknown	M	DF	Unknown	Unknown	Unknown	Unknown
752	2.34	921	7.14	High	0.9	Late	3	21	F	Puebla	Free Union	High School	Unemployed	Heterosexual

753	5.15	241	7.17	High	0.37	Recent	3	32	F	Tijuana	Single	High School	Employed	Heterosexual
754	3.67	393	7.17	High	0.64	ND	3	26	M	Edo. Méx	Unknown	Unknown	Unknown	Unknown
755	5.47	137	7.18	High	0.2	ND	3	26	M	DF	Unknown	Unknown	Unknown	Unknown
756	3.78	314	7.18	High	0.29	Late	3	30	M	Tijuana	Single	High School	Unemployed	MSM
757	4.92	326	7.22	High	0.33	ND	3	26	M	Edo. Méx	Unknown	Unknown	Unknown	Unknown
758	4.71	270	7.23	High	0.37	ND	2	23	M	Jalisco	Unknown	Unknown	Unknown	Unknown
759	4.15	878	7.26	High	0.67	ND	2	43	M	Edo. Méx	Unknown	Unknown	Unknown	Unknown
760	3.82	436	7.28	High	0.44	ND	3	25	M	Morelos	Unknown	Unknown	Unknown	Unknown
761	4.68	324	7.31	High	0.39	ND	2	24	M	Edo. Méx	Unknown	Unknown	Unknown	Unknown
762	5.18	441	7.32	High	0.23	ND	3	23	F	Guerrero	Unknown	Unknown	Unknown	Unknown
763	4.67	352	7.33	High	0.28	ND	3	23	M	Edo. Méx	Unknown	Unknown	Unknown	Unknown
764	5.20	609	7.34	High	0.52	ND	3	27	M	DF	Unknown	Unknown	Unknown	Unknown
765	5.23	281	7.36	High	0.21	ND	3	24	M	Edo. Méx	Unknown	Unknown	Unknown	Unknown
766	4.50	181	7.37	High	0.46	ND	3	28	M	Oaxaca	Unknown	Unknown	Unknown	Unknown
767	4.33	417	7.49	High	0.35	ND	ND	21	M	Morelos	Unknown	Unknown	Unknown	Unknown
768	3.90	311	7.58	High	0.25	ND	ND	25	M	DF	Unknown	Unknown	Unknown	Unknown
769	4.63	341	7.87	High	0.27	ND	2	25	M	DF	Unknown	Unknown	Unknown	Unknown
770	4.57	111	8.05	High	0.09	ND	ND	40	M	Jalisco	Unknown	Unknown	Unknown	Unknown
