# Supplementary figures and images for: High HPgV replication is associated with improved surrogate markers of HIV progression
Source: PLoS One. 2017 Sep 14;12(9):e0184494. doi: 10.1371/journal.pone.0184494 (PMC5598987; doi:10.1371/journal.pone.0184494)

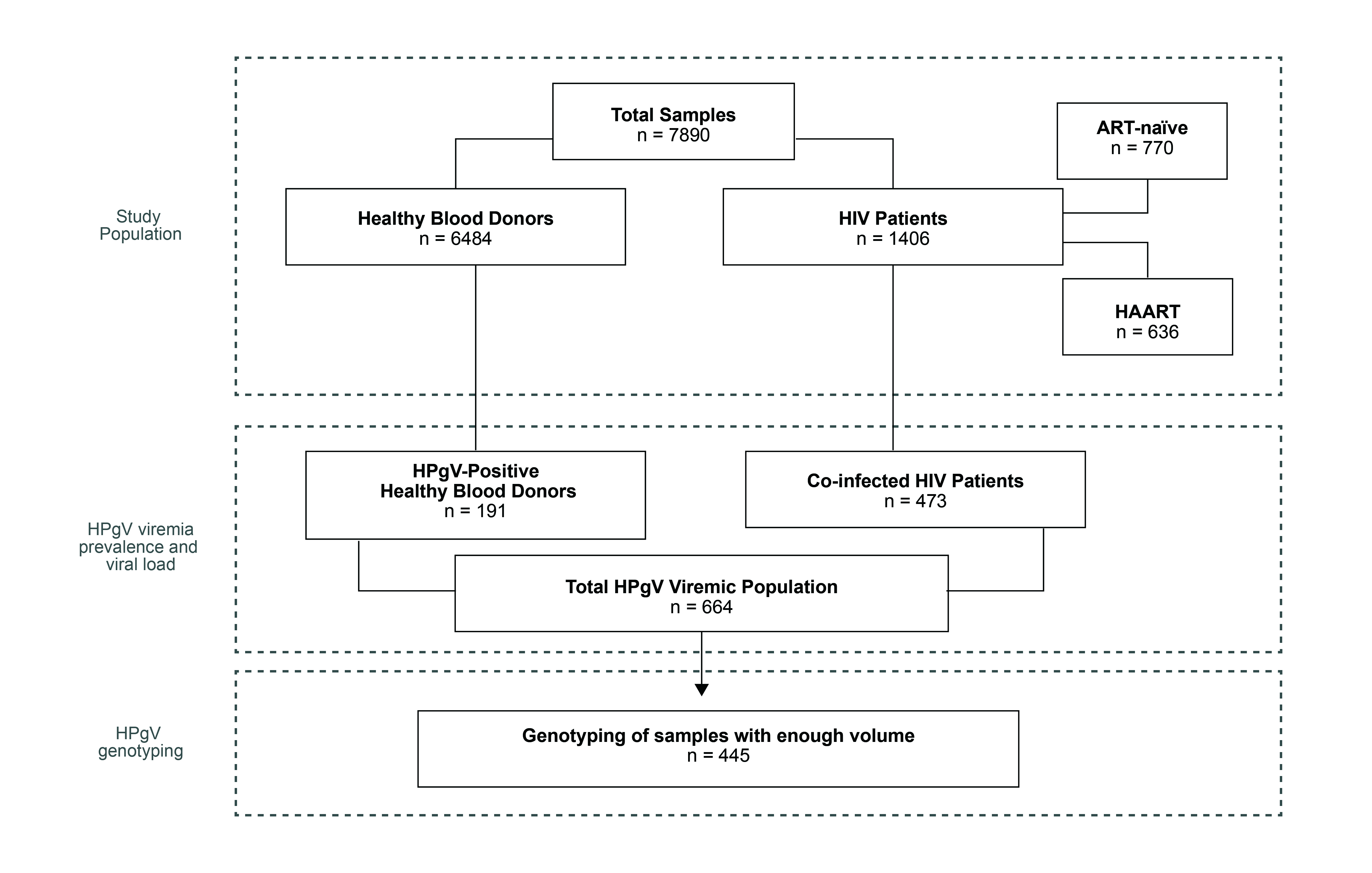

Supplement: S1 Fig — The flowchart shows the subset division of the samples used for HPgV viremia, viral load and genotype determination. (TIF) [file pone.0184494.s002.tif]

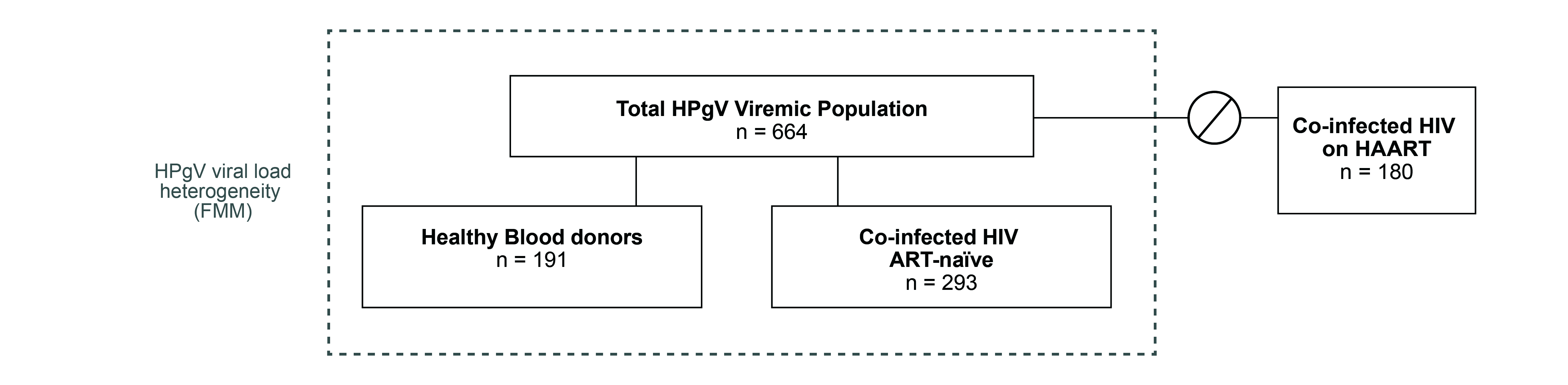

Supplement: S2 Fig — FMM analysis of HPgV viral load heterogeneity in healthy blood donors and co-infected ART-naïve patients. The co-infected HIV patients in HAART were not included in this analysis. (TIF) [file pone.0184494.s003.tif]

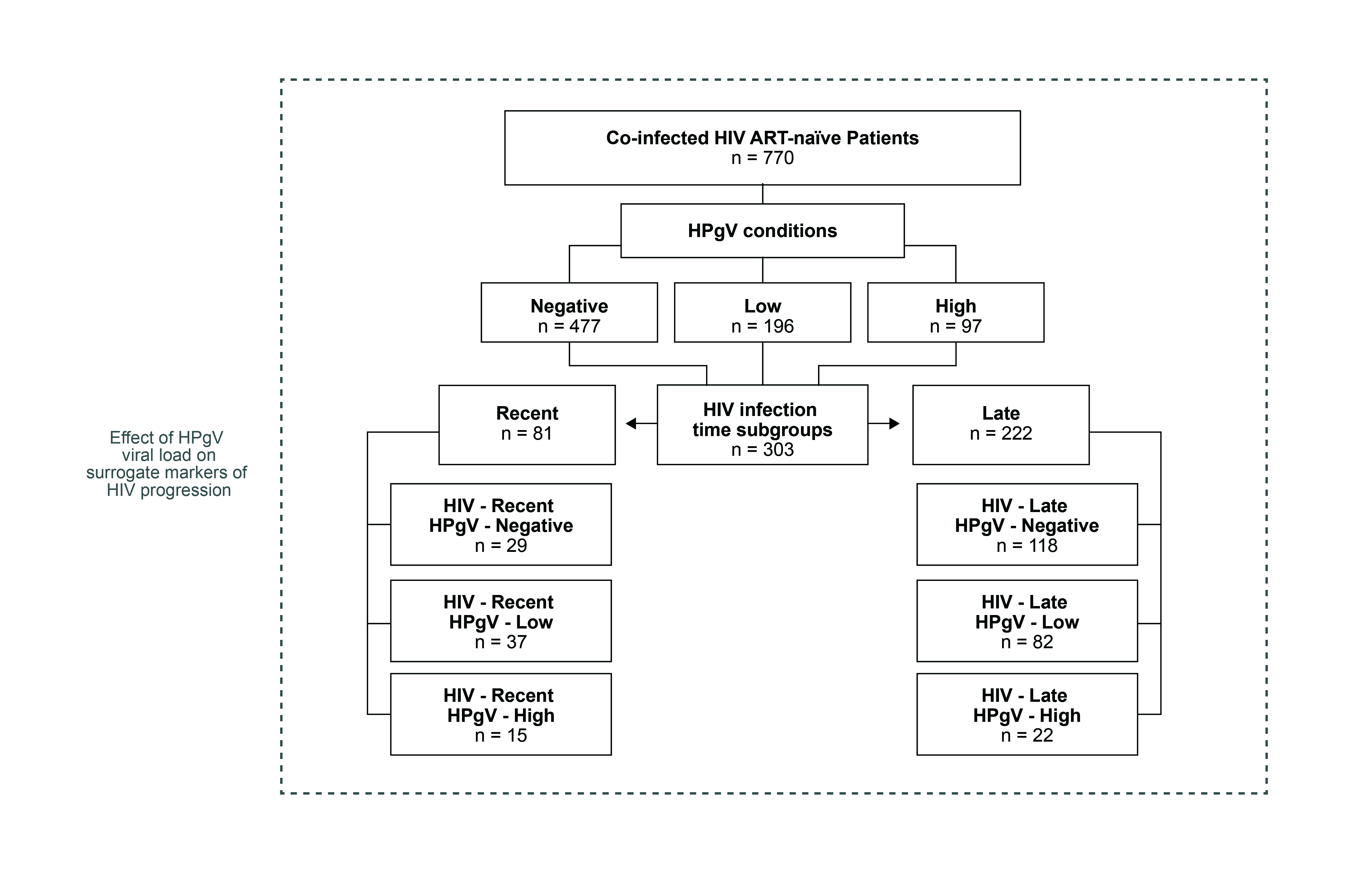

Supplement: S3 Fig — The flowchart shows the subsets division of the samples according to HPgV conditions and HIV infection time. (TIF) [file pone.0184494.s004.tif]

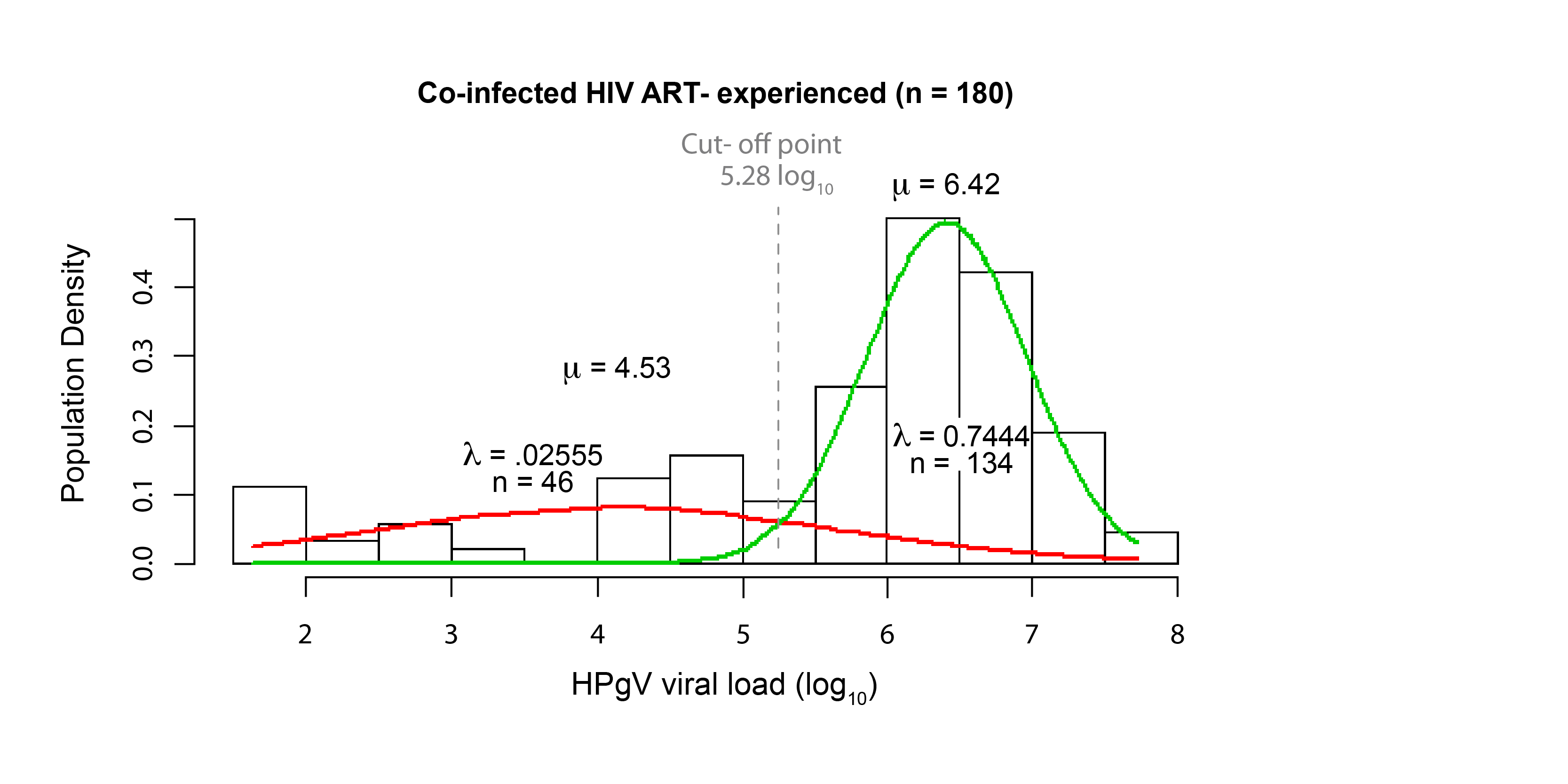

Supplement: S4 Fig — Density distribution (bars) of HPgV viral load (log10 genome equivalents) from co-infected ART-experienced patients. Gaussian curves indicate the fitted low (red line), high (green line), mean component curves (μ), the number of patients (n) and proportion (λ) in each population component. The clusters’ cut-off point is the intersection of the curves. (TIF) [file pone.0184494.s005.tif]

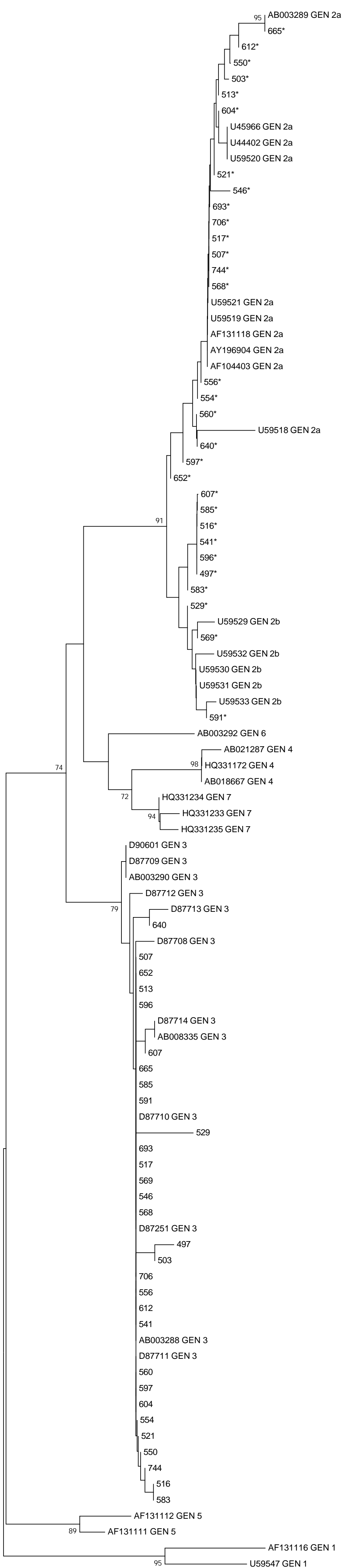

0.02

Supplement: S5 Fig — The phylogenetic analysis was constructed from partial nucleotide sequences of 5’UTR region by using the neighbor-joining method with MEGA software version 6. Reference strains were selected from GenBank and included. Bootstrap values are percentages of 1000 iterations. Reference strains were labelled as follows: GenBank accession number + word “GEN” + corresponding HPgV genotype, e.g. AB003289 GEN 2a. Patients samples are identified by Patient ID* for genotypes 2 and Patient ID for genotypes 3. (PDF) [file pone.0184494.s006.pdf]
